# Foetal growth in pregnant women with HIV

**DOI:** 10.1097/QAD.0000000000004294

**Published:** 2025-07-08

**Authors:** Dhruv Darji, INTERBIO-21st Consortium, Shane Norris, Eric O. Ohuma, Joris Hemelaar

**Affiliations:** a Infectious Disease Epidemiology Unit, Nuffield Department of Population Health; b Nuffield Department of Women's & Reproductive Health; c Oxford Maternal & Perinatal Health Institute, Green Templeton College, University of Oxford, Oxford, UK; d MRC-Wits Developmental Pathways for Health Research Unit, University of the Witwatersrand, Johannesburg, South Africa; e School of Human Development and Health, University of Southampton, Southampton; f Centre for Maternal, Adolescent, Reproductive, and Child Health, London School of Hygiene & Tropical Medicine, London, UK.

**Keywords:** abdominal circumference, biparietal diameter, estimated foetal weight, femur length, foetal growth, head circumference, HIV, small for gestational age, ultrasound

## Abstract

**Objective:**

Pregnant women with HIV (PWHIV) are at increased risk of delivering babies that are small for gestational age (SGA). We aimed to determine the foetal growth trajectories of PWHIV, compared to HIV-negative women.

**Design:**

Prospective pregnancy cohort study in Soweto, South Africa, in 2013–2016.

**Methods:**

Serial ultrasound measurements (every 5 ± 1 weeks) of foetal head circumference, biparietal diameter (BPD), abdominal circumference and femur length were obtained from less than 14 weeks’ gestation to term. Multivariable linear mixed effects models were used to estimate differences in mean foetal growth measures [head circumference, BPD, abdominal circumference and femur length, and estimated foetal weight (EFW)] and mean foetal growth velocity increments (head circumference, BPD, abdominal circumference, and femur length) according to maternal HIV status. *Z*-scores and centiles were calculated according to the INTERGROWTH-21^st^ standards for foetal growth. Multivariable mixed effects logistic regression was used to examine the association of maternal HIV infection with in-utero SGA (EFW <10^th^ centile) and very SGA (VSGA, EFW <3^rd^ centile).

**Results:**

Ultrasound measurements of 228 PWHIV and 384 HIV-negative pregnant women, with a median of five antenatal ultrasound scans per women, were analysed. There were no significant differences in mean foetal growth measures of head circumference [−0.37 mm, 95% confidence interval (CI) −1.46 to 0.72], BPD (0.07 mm, 95% CI −0.45 to 0.30), abdominal circumference (0.15 mm, 95% CI −1.47 to 1.17), femur length (0.04 mm, 95% CI −0.27 to 0.34) and EFW (1 g, 95% CI 0.98–1.02), and growth velocity increments for head circumference (−0.07 mm/week, 95% CI −0.24 to 0.10), BPD (−0.01 mm/week, 95% CI −0.06 to 0.04), abdominal circumference (0.19 mm/week, 95% CI −0.06 to 0.44) and femur length (0.00 mm/week, 95% CI −0.04 to 0.04) between foetuses of PWHIV and HIV-negative women. The prevalence of SGA ranged between 14.2% and 26.4% for PWHIV and between 18.5% and 24.1% for HIV-negative pregnant women during different gestation windows. There was no significant association between maternal HIV infection and in-utero SGA [adjusted odds ratio (aOR) 0.90, 95% CI 0.54–1.52] or VSGA (aOR 1.16, 95% CI 0.55–1.54). A sensitivity analysis restricted to PWHIV who received efavirenz-based antiretroviral therapy (ART) yielded results consistent with the overall analysis.

**Conclusion:**

Maternal HIV infection treated with ART does not appear to be associated with altered foetal growth, foetal growth velocity, or in-utero SGA, compared to HIV-negative women. Our findings support current international clinical guidelines recommending ART for PWHIV to improve maternal health and reduce vertical HIV transmission.

## Introduction

In 2023, 39.9 million people were living with HIV globally, with most of the burden concentrated in sub-Saharan Africa (SSA). Women and girls account for 53% of all people living with HIV; in SSA, they suffer 62% of all new HIV infections with an estimated 3100 infections per week amongst women aged 15–24 [1]. Annually, 1.2 million women with HIV become pregnant, 91% of whom reside in SSA [1]. Amongst these, 84% of pregnant women with HIV (PWHIV) receive antiretroviral therapy (ART) [1]. SSA also has the highest rates of neonatal and child mortality [2]. Newborns who are preterm, small for gestational age (SGA) or have low birthweight account for most neonatal deaths [2,3]. SGA is defined as an estimated foetal weight or birth weight below the 10^th^ centile, corrected for gestational age, compared to a reference standard [4,5]. In SSA, SGA contributes to approximately 21.9% of neonatal deaths [6]. The United Nations’ Sustainable Development Goal 3 aspires to decrease neonatal and under-5 mortality to 12 and 25 per 1000 live births, respectively, by 2030 [7]. However, the majority of countries in SSA are not on track to achieve these goals [8]. Given the overlapping epidemics of HIV and neonatal and child mortality, it is imperative to understand better the association between maternal HIV infection and adverse perinatal outcomes, such as SGA.

Untreated maternal HIV infection is associated with an increased risk of SGA, compared with HIV-negative mothers [9]. Current guidelines recommend the use of ART for PWHIV to improve maternal health and reduce vertical HIV transmission [1,10]. However, ART does not eliminate the SGA risk, as PWHIV receiving ART (comprising any triple drug ART regimen) have an increased risk of SGA, compared to PWHIV not on ART and HIV-negative women [11]. Hence, the dual burden of maternal HIV infection and ART contribute towards the risk of SGA amongst PWHIV [11]. The risk of SGA amongst PWHIV on ART may also differ based on the type of ART, as protease inhibitor-based ART is associated with a higher risk of SGA compared to nonnucleoside reverse transcriptase inhibitor (NNRTI)-based ART [12]. It has been estimated that more than two million SGA newborns in SSA were attributable to maternal HIV infection and ART use over the past three decades [13].

Although the risk of SGA at birth associated with maternal HIV and ART has been assessed in many studies, how intra-uterine foetal growth leads to SGA at birth remains an underexplored area of HIV perinatal epidemiology. Understanding the impact of maternal HIV infection and ART on foetal growth may highlight opportunities for intervention and help improve perinatal outcomes. The few studies that have assessed foetal growth in PWHIV report no overall difference in growth parameters amongst foetuses of PWHIV on ART compared to HIV-negative women [14–18]. However, these studies had several important limitations including limited power [14,15,18,19], inaccurate gestational age dating [16–18], inappropriate controls and selection bias [14,15,17], limited numbers of ultrasound measurements per participant [16–19] and high loss to follow-up [14]. Hence, the association of maternal HIV infection and ART with foetal growth remains uncertain.

The present study, therefore, aimed to assess the association of maternal HIV infection and ART with foetal growth measures [head circumference, abdominal circumference, biparietal diameter (BPD), femur length, and estimated foetal weight (EFW)] and foetal growth velocity increments of head circumference, abdominal circumference, BPD and femur length, based on five-weekly ultrasound scans of pregnant women with accurate first-trimester estimation of gestational age in South Africa.

## Methods

### Study design

A prospective pregnancy cohort study was conducted at the Chris Hani Baragwanath Academic Hospital in Soweto, South Africa between 2013 and 2016 [20]. All participants were enrolled at a gestational age of less than 14 weeks, and baseline information was obtained from their medical records, antenatal cards and questionnaire-based interviews. Gestational age was determined by the last menstrual period (LMP) and confirmed with a dating ultrasound scan at less than 14 weeks, performed by ultrasonographers trained to measure foetal crown-rump length. Only women with ultrasound-confirmed gestational age less than 14 weeks were included in the study. Women were excluded from the study if there was a discrepancy between LMP and CRL-based gestational age of more than 7 days. Women were then seen every 5 ± 1 weeks (approximately at 14–18, 19–23, 24–28, 29–33, 34–38, and ≥ 39 weeks) for antenatal assessments and detailed foetal ultrasound scans. At each follow-up visit, trained ultrasonographers conducted transabdominal ultrasound scans recording four foetal size measurements (head circumference, BPD, abdominal circumference and femur length) using a Philips HD-9, Philips Ultrasound machine (Bothell, Washington, USA). Each parameter was measured in triplicate, and all ultrasound images underwent quality rating by blinded external assessors at the University of Oxford using an image-scoring scheme for foetal ultrasound images described by Salomon *et al.* [21].

### Participant characteristics

The study enrolled 680 black South African pregnant women living in Soweto, aged at least 18 years, with a singleton pregnancy. Women were excluded if they had a multiple pregnancy, BMI greater than 35 kg/m^2^ or intellectual/physical disability [20]. All women not known to be HIV-positive were offered an HIV test at enrolment and every follow-up visit. Detailed information on maternal HIV diagnosis and ART use was captured from medical records and confirmed by direct interviews. PWHIV were defined as participants with documentation of an HIV-positive laboratory test result either at baseline, during any pregnancy follow-up visit, or at delivery. At the time of the study, CHBAH implemented the South African ART guidelines, which recommended tenofovir disoproxil fumarate (TDF), emtricitabine (FTC)/lamivudine (3TC) and efavirenz (EFV) for all PWHIV [22,23].

### Outcomes

The primary outcomes were mean foetal growth measures of head circumference, BPD, abdominal circumference, femur length (in millimetres) and EFW (in grams) and mean growth velocity increments for head circumference, BPD, abdominal circumference and femur length (in millimetres/week). In-utero SGA and VSGA were the secondary outcomes.

Estimated foetal weight (EFW) was calculated using the three-parameter formula reported by Hadlock *et al.* [24].


$$\text{EFW}=10^{1.326+\left(0.0107\times \text{HC}\right)+\left(0.0438\times \text{AC}\right)+\left(0.158\times \text{FL}\right)+\left(-0.00326\times \text{AC}\times \text{FL}\right)}$$

Where EFW is expressed in grams and abdominal circumference (AC), head circumference (HC) and femur length (FL) are in centimetres.

Growth velocity increments [25], describing the increment in growth measures per week, were calculated as follows:


$$\text{Velocity}\;\text{increment}=\frac{\left(Y_{2}-Y_{1}\right)}{\left(t_{2}-t_{1}\right)}$$

Where *Y*_2_ and *Y*_1_ (in millimetres) are two consecutive ultrasound measurements, and *t*_2_ and *t*_1_ (completed weeks) are the respective gestational ages when the measurements were taken.

*Z*-scores and centiles for all parameters were calculated according to the INTERGROWTH-21^st^ standards for foetal growth [5,25,26]. Outliers, defined as measurements with *z*-scores greater than 5 SD or less than −5 SD at each gestational age, were excluded from analyses (Supplementary Table 14). SGA was defined as EFW below the 10^th^ centile for gestational age.

### Covariates

Our literature search identified factors associated with foetal growth and maternal HIV infection from which we determined *a priori* the set of potential confounders to be adjusted for in the multivariable analyses (Supplementary Figure 1). These included maternal age, maternal BMI (baseline BMI, categorized according to the WHO classification), smoking, alcohol, parity, marital status, socio-economic indicators (occupation, education status and wealth index score), history of adverse perinatal outcomes (history of low birthweight, preterm birth, stillbirth or neonatal death) and newborn sex [14,15,20,27].

### Statistical analyses

Data were available for 633 participants enrolled in the study (Supplementary Figure 2). Participants with missing foetal biometry data (*n* = 21, 3.3%) were missing at random and were excluded from further analyses. Linear mixed effects models (LMM) were used to analyse the associations between maternal HIV status and foetal growth outcomes, accounting for repeated and clustered ultrasound measurements (Supplementary Table 13). To model trajectories of foetal growth parameters using LMM, gestational age terms were specified as fixed effects. The best-fitting powers of gestational age for each growth parameter were determined using reference interval estimation by generalized least squares with fractional polynomials [28]. A random intercept for each participant (capturing baseline variability) and a random slope for gestational age (allowing a variable effect of gestational age on foetal growth between participants) were specified. Covariance structures were compared using Akaike Information Criteria (AIC) to determine the most suitable for each LMM. Maternal HIV status was added as a fixed effect in LMMs describing each growth parameter. Univariable and multivariable LMMs, adjusting for the listed confounders, were fitted for all the foetal growth outcomes. EFW was log-transformed (natural logarithm) to address skewness. The resulting EFW estimates were geometric means, derived by exponentiating the coefficients and confidence intervals (CI) of the log-transformed outputs. To avoid retransformation bias in the exponentiated estimates of EFW, back-transformed means were corrected with the variance of residuals in the LMM [29]. LMM assumptions were tested by assessing the distribution of residuals (histograms and quantile-quantile plots) and variance of residuals (residuals versus fitted plots) in the final models. Marginal means for each exposure were predicted at midpoints of the follow-up time windows during pregnancy. Mean differences (and geometric mean difference for EFW) at these gestational ages were calculated by maternal HIV status. Gestational age was determined *a priori* as an effect modifier, and a likelihood ratio test for interaction was performed between maternal HIV status and gestational age in all fully adjusted LMMs.

Multivariable mixed effects logistic regression was used to analyse the association of maternal HIV infection with in-utero SGA and VSGA. In this model, gestational age (categorized according to follow-up time windows) was added as a confounder. In addition, stratified analysis of the association was done by gestational age categories. The ‘34–38 weeks’ and ‘≥39 weeks’ gestational age categories were combined into one category (‘≥34 weeks’) due to the low number of outcomes in the latter group. A likelihood ratio test for heterogeneity was used to assess if the odds ratios (ORs) were the same across different gestational age intervals.

### Sensitivity analyses

The LMM analyses were re-run after restricting analysis to ultrasound images with the highest quality ratings. As a supplementary analysis using birth weight data, newborns were classified as SGA and VSGA if the birthweight for gestational age and sex was less than the 10^th^ and 3^rd^ centile (with reference to INTERGROWTH-21^st^ newborn standard [4]), respectively, and checked for associations with maternal HIV infection using multivariable logistic regression. Foetal growth analyses were restricted to PWHIV known to be treated with EFV-based ART during pregnancy to compare these findings to the main results from the complete cohort of PWHIV. To assess differences based on the timing of ART initiation, we conducted a sensitivity analysis to compare foetal growth amongst PWHIV who initiated ART preconception versus antenatally. A two-tailed *P* less than 0.05 was considered statistically significant. All statistical analyses were performed in Stata (version 18).

### Ethical approval

The study was approved by the University of Oxford Tropical Research Ethics Committee (reference 1008-13) and the Human Research Ethics Committee (Medical) of the University of the Witwatersrand, Johannesburg, South Africa (M240164).

## Results

A total of 612 women were included in the analyses: 228 (37.3%) PWHIV and 384 (62.7%) HIV-negative women (Supplementary Figure 2). PWHIV were older than HIV-negative women (*P* < 0.001) (Supplementary Table 1). More HIV-negative women (18.4%) were nulliparous than PWHIV (12.7%). A higher proportion of HIV-negative women (43.2%) reported baseline history of adverse perinatal outcomes compared to PWHIV (32.1%) (Supplementary Table 1).

HIV diagnosis dates were available for 106 (46.5%) PWHIV, of whom 76 (71.7%) were diagnosed preconception and 30 (28.3%) antenatally. Among those diagnosed antenatally, 96.7% were identified at their first antenatal visit at a median gestational age of 8 weeks [interquartile range (IQR) 6–10.3 weeks] (Supplementary Table 2). ART information was available for 121 (53.1%) PWHIV, of whom 119 (98.3%) received ART during pregnancy, nearly all of whom (94.1%) received EFV-based ART (Supplementary Table 3). Timing of ART initiation was known for 110 (48.2%) of PWHIV, with 40 (36.4%) initiating ART preconception and 70 (63.6%) antenatally (Supplementary Table 3). Of the 218 livebirths to PWHIV, 216 (99.1%) were HIV-negative and HIV-status was unknown for two. There were 99 (17.2%) preterm births in the study, 55 (15.4%) amongst HIV-negative women and 44 (20.2%) amongst PWHIV (*P* = 0.15) (Supplementary Table 4).

The median number of follow-up scans was five (IQR 4–5) in both PWHIV and HIV-negative women (Supplementary Table 5). Of the 612 participants, 554 (90.5%) were seen for antenatal scans between 14 and 18 weeks, 571 (93.3%) between 19 and 23 weeks, 537 (93.6%) between 24 and 28 weeks, 498 (81.3%) between 29 and 33 weeks and 405 (66.2%) between 34 and 38 weeks (Supplementary Table 5).

**Fig. 1:**
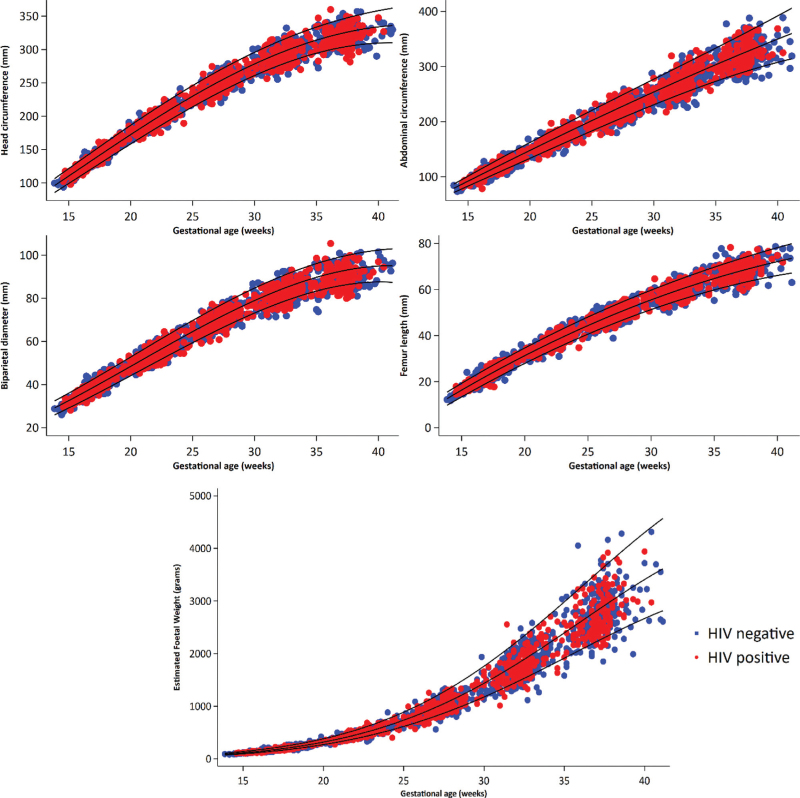
Distribution of foetal growth measures during pregnancy by maternal HIV status.

The distribution of foetal growth measures (Fig. 1) and velocity increments (Supplementary Figure 3) throughout pregnancy are shown. The unadjusted median *z*-scores for head circumference, BPD, abdominal circumference, femur length and EFW were generally less than zero with no statistically significant differences between the exposure groups during any of the gestational age windows (Supplementary Table 5). Unadjusted LMM showed no significant differences in the mean head circumference (*P* = 0.75), BPD (*P* = 0.61), abdominal circumference (*P* = 0.83) and femur length (*P* = 0.86) between foetuses of PWHIV and HIV-negative women during pregnancy (Supplementary Table 6.1).

Dark lines representing 3^rd^ (bottom), 50^th^ (middle) and 97^th^ (top) centiles for respective foetal growth measures from the INTERGROWTH-21^st^ foetal growth standards.

**Fig. 2:**
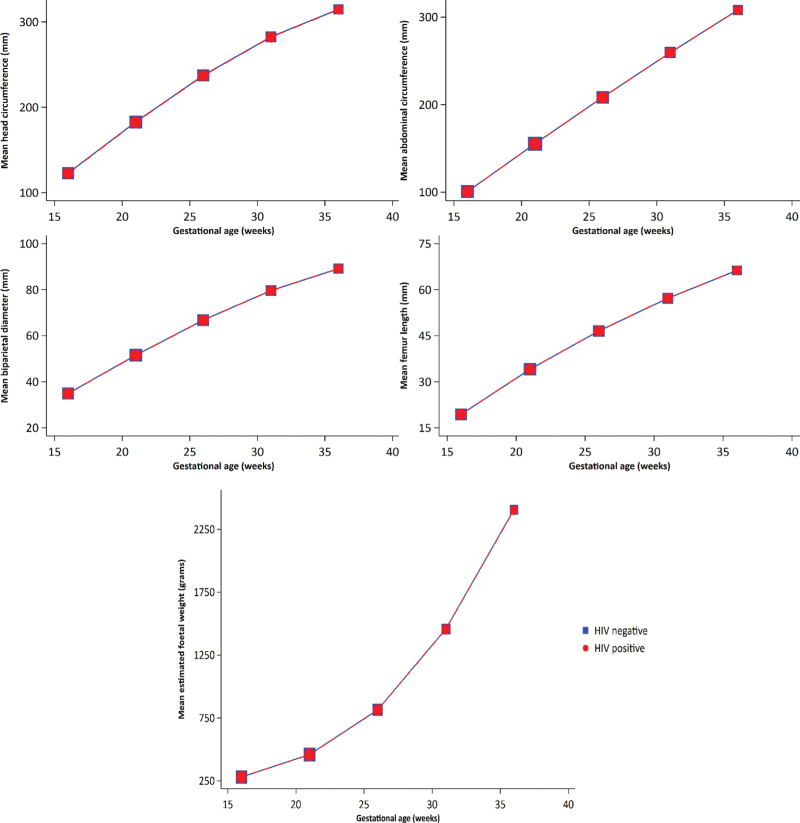
Adjusted mean growth measures by maternal HIV status.

Figure 2 shows the means of foetal growth measures by maternal HIV status after adjusting for maternal age, baseline BMI, smoking, alcohol, parity, marital status, occupation, education, wealth index score, history of adverse perinatal outcomes and foetal sex. There were no statistically significant differences in the adjusted mean head circumference (−0.37 mm, 95% CI −1.46 to 0.72), mean BPD (0.07 mm, 95% CI −0.45 to 0.30), mean abdominal circumference (0.15 mm, 95% CI −1.47 to 1.17) and mean femur length (0.04 mm, 95% CI −0.27 to 0.34) between foetuses of PWHIV and HIV-negative women (Fig. 2, Supplementary Table 6.2). Tests for interaction showed no significant differences in the association of maternal HIV infection with head circumference (*P*_int_ = 0.25), BPD (*P*_int_ = 0.26), abdominal circumference (*P*_int_ = 0.58) and femur length (*P*_int_ = 0.53) by gestational age. There was no significant difference in the adjusted mean EFW between foetuses of PWHIV and HIV-negative women (1 g, 95% CI 0.98–1.02) (Fig. 2, Supplementary Table 7.2). The differences in mean EFW between the two exposure groups did not significantly vary during pregnancy (*P*_int_ = 0.49). The highest quality ratings were received for 68.7% head circumference, 68.7% BPD, 73.6% abdominal circumference and 78.9% femur length images. Restricting analyses to these data did not change the LMM results, as no significant mean differences in foetal growth measures or velocity increments were seen between PWHIV and HIV-negative women (Supplementary Table 8).

Means derived from multivariable linear mixed effects models adjusted for maternal age, baseline BMI, smoking, alcohol, parity, marital status, occupation, education, wealth index score, history of adverse perinatal outcomes and foetal sex. Predicted estimated foetal weight values are geometric means.

**Fig. 3:**
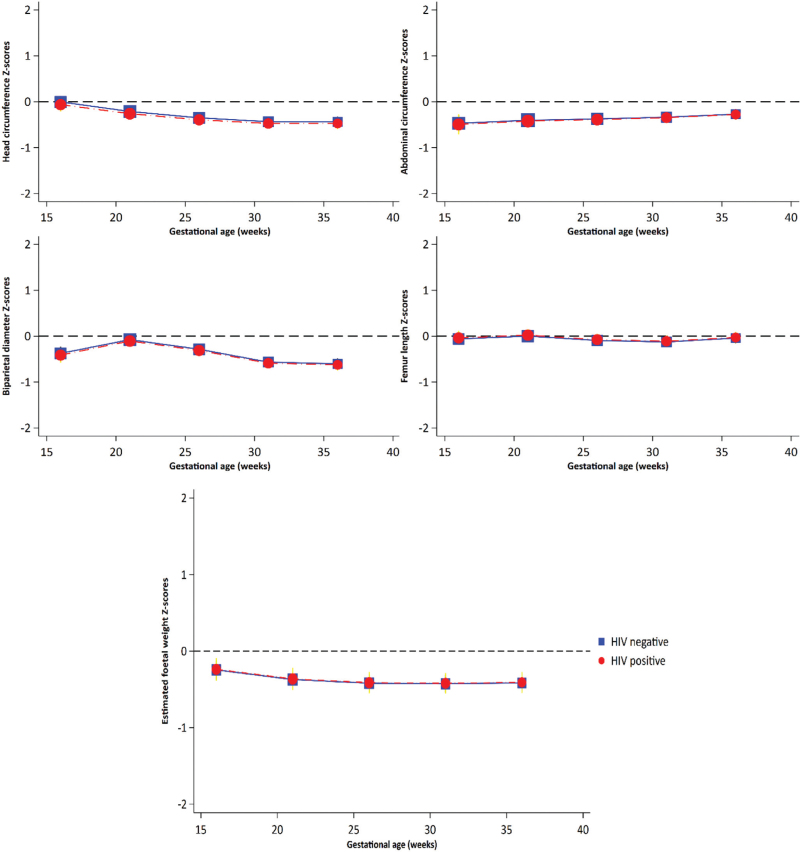
*Z*-scores of adjusted mean growth measures by maternal HIV status.

We calculated the *z*-scores of the predicted means from all LMMs, after adjusting for confounders, to compare foetal growth parameters in our cohort to the INTERGROWTH-21^st^ foetal reference standards. The *z*-scores of predicted mean growth measures at different gestational ages ranged between −1 and 0 for both foetuses of PWHIV and HIV-negative women (Fig. 3, Supplementary Table 5).

Z-scores of mean growth measures calculated with reference to INTERGROWTH-21^st^ foetal standards. Means derived from multivariable linear mixed effects models adjusted for maternal age, baseline BMI, smoking, alcohol, parity, marital status, occupation, education, wealth index score, history of adverse perinatal outcomes and foetal sex. Estimated foetal weight *z*-scores are calculated from predicted geometric means.

**Fig. 4:**
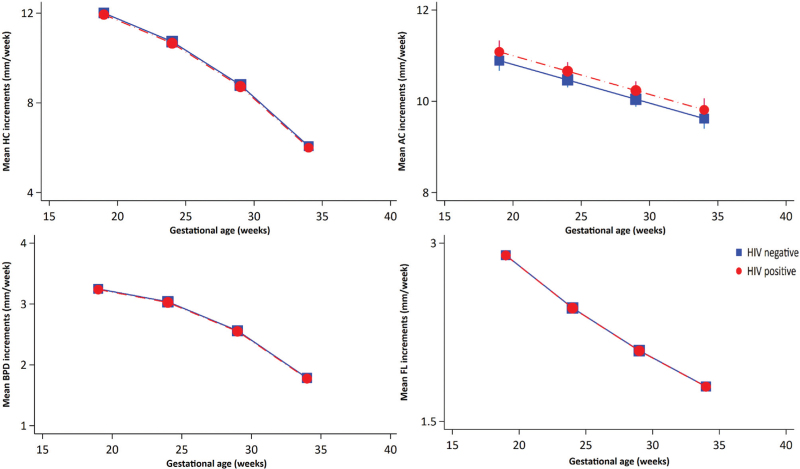
Adjusted mean growth velocity increments by maternal HIV status.

We studied growth velocity increments to assess differences in the rate of foetal growth by maternal HIV status. Unadjusted mean growth velocity increments did not differ significantly by maternal HIV status (Supplementary Table 7.1). Figure 4 shows the mean growth velocity increments for head circumference, BPD, abdominal circumference and femur length, adjusted for confounders, according to maternal HIV status. There were no statistically significant differences in growth velocity increments for head circumference (−0.07 mm/week, 95% CI −0.24 to 0.10), BPD (−0.01 mm/week, 95% CI −0.06 to 0.04), abdominal circumference (0.19 mm/week, 95% CI −0.06 to 0.44) and femur length (0.00 mm/week, 95% CI −0.04 to 0.04) between foetuses of PWHIV and HIV-negative women (Supplementary Table 7.2). There was no significant interaction with gestational age [head circumference velocity increments (*P*_int_ = 0.92), BPD velocity increments (*P*_int_ = 0.10), abdominal circumference velocity increments (*P*_int_ = 0.22) and femur length velocity increments (*P*_int_ = 0.28)]. For predicted mean growth velocity increments, all *z*-scores were between −1 and 0 for foetuses of both PWHIV and HIV-negative women, except femur length velocity increments *z*-scores, which were greater than 0 towards the end of pregnancy (Supplementary Figure 4 and Supplementary Table 7.2).

Mean growth velocity increments derived from multivariable linear mixed effects models adjusted for maternal age, baseline BMI, smoking, alcohol, parity, marital status, occupation, education, wealth index score, history of adverse perinatal outcomes and foetal sex. AC, abdominal circumference; BPD, biparietal diameter; FL, femur length; HC, head circumference.

**Fig. 5:**
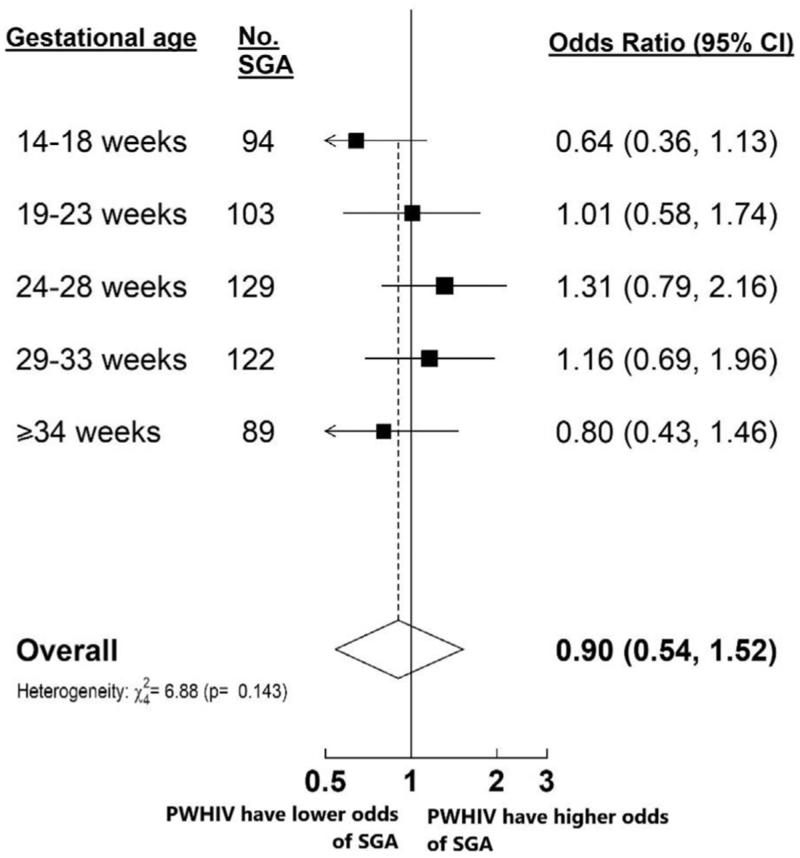
Association of maternal HIV infection with in-utero small-for-gestational age stratified by gestational ages.

The prevalence of in-utero SGA ranged between 14.2% and 26.4% for PWHIV and 18.5% and 24.1% for HIV-negative women during different gestation windows and peaked at 24–28 weeks and 29–33 weeks’ gestation, respectively (Supplementary Table 5). The odds of a foetus being SGA were not significantly different in PWHIV compared to HIV-negative women, after adjusting for the aforementioned confounders and gestational age (aOR 0.90, 95% CI 0.54–1.52) (Fig. 5). Stratified analyses showed no significant differences in the association of maternal HIV status and in-utero SGA during the different gestational windows (*P*_heterogeneity_ = 0.14, Fig. 5). The odds of the foetus of a PWHIV being VSGA were also not significantly different compared to HIV-negative women, after adjusting for confounders and gestational age (aOR 1.16, 95% CI 0.55–2.42) (Supplementary Figure 5). Moreover, there was no significant association between maternal HIV infection and in-utero SGA (aOR 0.70, 95% CI 0.44–1.11) or VSGA (aOR 0.69, 95% CI 0.35–1.30) at the last ultrasound scan before delivery (Supplementary Table 9). We analysed birthweight data in the same cohort and found that the odds of newborns of PWHIV being SGA at birth (aOR 1.56, 95% CI 0.93–2.62) or VSGA at birth (OR 1.78, 95% CI 0.69–4.61) were not significantly different compared to HIV-negative women, after adjusting for confounders (Supplementary Table 10). Restricting foetal growth analyses to PWHIV receiving EFV-based ART showed no significant differences in foetal growth measures compared to HIV-negative women (Supplementary Figures 6 and 7, Supplementary Table 11). Finally, comparing foetal growth measures between PWHIV who initiated ART preconception versus antenatally did not show any significant differences (Supplementary Table 12). These findings are consistent with the foetal growth analyses conducted on the complete cohort of PWHIV, where ART exposure during pregnancy was known for only half of the participants.

SGA defined as estimated foetal weight (EFW) below 10^th^ centile with reference to INTERGROWTH-21^st^ EFW standards. Adjusted odds ratios derived from multivariable mixed effects logistic regression model adjusted for maternal age, baseline BMI, smoking, alcohol, parity, marital status, occupation, education, wealth index score, history of adverse perinatal outcomes, foetal sex and gestational age. CI, confidence interval; SGA, small-for-gestational-age.

## Discussion

We found no differences in the mean head circumference, BPD, abdominal circumference, femur length, and EFW measures and growth velocity increments of head circumference, BPD, abdominal circumference and femur length between foetuses of PWHIV and HIV-negative women. There was no association between maternal HIV status and risk of a foetus being SGA or VSGA *in utero*. The lack of association of maternal HIV status with foetal growth measures, growth velocity increments and SGA/VSGA, was seen across pregnancy. Among the 53.1% of PWHIV whose ART use was known, 94.1% were on EFV-based ART. Restricting the foetal growth analyses to women who received EFV-based ART showed results consistent with those from the complete cohort of PWHIV. Considering these findings, along with no recorded cases of mother-to-child transmission of HIV among the PWHIV in our study, as well as local guidelines, which recommended EFV-based ART for all PWHIV during the study period [22,23], we believe it is reasonable to interpret our results as pertaining to PWHIV who were mostly on EFV-based ART.

Our results broadly align with the existing evidence on the association of maternal HIV infection and ART use with foetal growth. A small cohort study in South Africa with 122 PWHIV, all on EFV-based ART, showed no overall differences in mean foetal sizes and growth velocities between PWHIV and HIV-negative women, as shown in our results [14]. The study reported that HIV-exposed female foetuses had smaller head circumference, while HIV-exposed males had larger femur length, based on stratified analyses by placental morphology and foetal sex. However, this study could have been underpowered with potential selection bias due to exclusion of 63% of participants. Further, the results of subgroup analyses by foetal sex were not corrected for multiple testing and could be due to chance. A mixed Brazilian/US cohort of 166 PWHIV [all on ART, including NNRTI-based, PI-based, and integrase strand transfer inhibitor (INSTI)-based regimens with a TDF-3TC backbone] and 705 HIV-negative pregnant women found no overall differences in mean foetal growth measures by maternal HIV status [15]. They found early (<20 weeks’ gestation) lower mean *z*-scores for head circumference, BPD and abdominal circumference in PWHIV compared to HIV-negative women, with the differences diminishing by the end of pregnancy [15]. These results, suggesting that the effect of maternal HIV infection on foetal growth [particularly the skull measures (head circumference and BPD] was modified by gestational age, were not replicated in our study. The contrasting findings may be due to selection bias in the mixed cohort, which included HIV-negative controls from two studies where women were enrolled for being at high risk of Zika virus (ZIKV) infection, a known cause of microcephaly. Although they only included women uninfected with ZIKV, the controls were not selected independent of ZIKV infection risk, and undetected infections among HIV-negative participants could have biased the skull measurements. Other studies used fewer ultrasound measurements per participant and did not assess longitudinal foetal growth trajectories [18,19]. A study in Botswana found no significant difference in mean head circumference, BPD, abdominal circumference and femur length *z*-scores between 309 PWHIV [all on EFV-based or dolutegravir (DTG)-based ART with a TDF-3TC backbone] and 160 HIV-negative women based on a single measurement taken between 16 and 36 weeks’ gestation [16]. A South African study compared foetal long bone (femur length and humerus length) measures among 646 PWHIV based on duration of exposure to TDF-based ART and found no significant differences [17]. This study did not compare foetal biometry of PWHIV to HIV-negative women [17]. The mean *z*-scores in our study ranged between −1 and 0 for both PWHIV and HIV-negative women, indicating that foetuses in our cohort were generally smaller than the standards (INTERGROWTH-21^st^) [5]. This was consistent with other SSA studies using the same standards and may indicate a high burden of other risk factors for foetal growth abnormalities in these settings, possibly overshadowing the effects of maternal HIV and ART on foetal growth [14,16–18].

Our findings, along with other existing evidence on foetal growth [14–19], appear inconsistent with the current evidence of meta-analyses reporting an association of maternal HIV infection and ART use with an increased risk of SGA at birth, compared with HIV-negative women [9,11–13]. This discrepancy may stem from the lower statistical power of the fewer and smaller foetal growth studies compared with the larger meta-analyses of cohort studies on adverse perinatal outcomes in PWHIV, but other factors should be considered. Firstly, analyses of birthweight data in our cohort found no significant association between maternal HIV status and SGA or VSGA at birth; hence, our results are internally consistent. Secondly, the largest meta-analysis (19 observational studies) comparing PWHIV receiving ART with HIV-negative women, showed high heterogeneity (*I*^2^ 97.6%) in the pooled estimates, indicating divergent findings in individual studies [11]. Differences in newborn reference charts used to define SGA in the source studies may partly explain this, as different charts will lead to different classifications of neonates relative to specific cut-off points [30]. Thirdly, while meta-analyses have shown an association between PWHIV receiving any ART regimen and SGA, compared to HIV-negative women [11], comparisons between PWHIV receiving different ART regimens have shown that PWHIV receiving NNRTI-based ART (such as EFV-based ART) have a significantly lower risk of SGA and VSGA compared to PWHIV receiving protease inhibitor-based ART [12]. Hence, PWHIV receiving NNRTI-based ART may have a similar risk of SGA as HIV-negative women, as found in our study. Finally, the discrepancies may be due to relative differences between foetal growth standards and newborn size standards. Participants in the Foetal Growth Longitudinal Study (FGLS), which produced the INTERGROWTH-21^st^ foetal growth standards, were a selected subset of (very similar, but not identical to) the Newborn Cross-Sectional Study, which produced the INTERGROWTH-21^st^ newborn size standards [31]. This may be reflected in our cohort by the varying, though nonsignificant, point estimates for in-utero SGA (aOR 0.90, 95% CI 0.54–1.52) and at-birth SGA (aOR 1.56, 95% CI 0.93–2.62). In our study, in-utero SGA was defined using EFW calculated using the Hadlock formula, which is a composite of three separate measures [24]; hence, its precision may be influenced by ultrasound operator variability or relative foetal position. At-birth SGA was defined using physically measured birthweight, which is a more precise measure. Therefore, measurement error and differences in methods used to defined in-utero and at-birth SGA likely explain the variation in these findings.

Our study had several strengths. This study, to our knowledge, is the largest study to date on the association of maternal HIV infection with foetal growth, using 5-weekly serial ultrasound data from 612 pregnant women with an average of five scans per woman. Gestational age was determined by universal first trimester ultrasound, ensuring that accurate calculation of *z*-scores and centiles (used to define SGA/VSGA) and minimizing misclassification bias. To minimize random error, all ultrasound measurements were taken in triplicate by trained sonographers, and image quality was independently assessed. Sensitivity analyses based only on the highest quality images confirmed the findings of our main analyses.

Our study also had some limitations. Ultrasonographers were not blinded to participant information (including HIV status), potentially introducing measurement bias in the recordings. Our study was conducted during a period when EFV-based ART was the recommended first-line regimen for HIV treatment of pregnant women; hence, our results may not be generalizable to the current era of DTG-based ART as the preferred first-line regimen. The number of PWHIV included may have limited the study's statistical power to detect differences in foetal growth parameters or associations with SGA. Due to the observational nature of the study, residual confounding from unmeasured or imprecisely measured confounders cannot be eliminated despite adequate adjustment in the multivariable models. Information on timing of HIV diagnosis, ART use in pregnancy and timing of ART initiation was only available for 47%, 53% and 52% of PWHIV, respectively. This limited the study's power to analyse the impact of ART use as well as duration of HIV and ART exposure on foetal growth. We lacked information about HIV disease (stage of disease, CD4^+^ cell count or viral load) through which analyses by disease severity could be undertaken.

In summary, our findings indicate that in-utero foetal growth does not significantly differ between PWHIV receiving EFV-based ART and HIV-negative women, which is reassuring. Currently, the WHO recommends EFV-based ART as an alternative first-line regimen in adults and pregnant women and DTG-based ART as the preferred first-line ART regimen [10]. There is no evidence that the risk of SGA at birth differs between PWHIV receiving EFV-based ART compared to DTG-based ART.^12^ Future large studies with accurate gestational age dating and high-quality serial ultrasound measurements should evaluate the association of DTG-based ART with foetal growth.

## Acknowledgements

We thank all the study participants who took part in this study. We thank the doctors, nurses and staff at Chris Hani Baragwanath Academic Hospital (Department of Obstetrics and Gynaecology) who facilitated the study. We thank the ultrasonographers who performed the ultrasound scans and the study nurses who collected the data and weighted the newborns. We thank the Gates Foundation for funding this study (grant number 49038).

D.D. prepared the dataset for analyses, designed the statistical analyses plan, conducted the statistical analyses, created tables and figures, interpreted the findings and wrote the first draft of the manuscript. S.N. coordinated data collection. E.O. provided advice on the statistical analyses. J.H. conceived, designed and coordinated the study, designed the analysis plan, interpreted the findings, and wrote the manuscript. All authors read and approved the final version of the manuscript.

Data sharing statement: study data are available on reasonable request to the INTERBIO-21^st^ Consortium.

Funding: Gates Foundation.

### Conflicts of interest

There are no conflicts of interest.

## Supplementary Material

Supplemental Digital Content
