# Supplementary material for: Foetal growth in pregnant women with HIV
Source: AIDS. 2025 Jul 8;39(11):1568–79. doi: 10.1097/QAD.0000000000004294 (PMC12337926; doi:10.1097/QAD.0000000000004294)
Supplement: Supplemental Digital Content [file aids-39-1568-s001.docx]

**Foetal growth in pregnant women with HIV: Longitudinal analysis of serial ultrasound measurements in South Africa.**

**Supplementary Digital Content**

[Supplementary Figure 1. Directed acyclic graph of association of maternal HIV status with foetal growth. 2](#_Toc189429283)

[Supplementary Figure 2. Flow diagram of study participants 3](#_Toc189429284)

[Supplementary Figure 3. Distribution of foetal growth velocity increments during pregnancy by maternal HIV status 4](#_Toc189429285)

[Supplementary Figure 4. Z-scores of mean foetal growth velocity increments by maternal HIV status 5](#_Toc189429286)

[Supplementary Figure 5. Association of maternal HIV status with VSGA in-utero. 7](#_Toc189429287)

[Supplementary Figure 6: Adjusted mean growth measures for PWHIV who received EFV-based ART compared with HIV negative women. 8](#_Toc189429288)

[Supplementary Figure 7: Z-scores of adjusted mean growth measures for PWHIV who received EFV-based ART compared with HIV negative women. 10](#_Toc189429289)

[Supplementary Table 1. Baseline characteristics of participants by HIV status. 12](#_Toc189429290)

[Supplementary Table 2. Timing of HIV diagnosis 14](#_Toc189429291)

[Supplementary Table 3. Summary of ART use during pregnancy 15](#_Toc189429292)

[Supplementary Table 4. Foetal and newborn characteristics by maternal HIV status 16](#_Toc189429293)

[Supplementary Table 5. Summary of antenatal follow up and unadjusted foetal growth z-scores and prevalence of SGA and VSGA. 18](#_Toc189429294)

[Supplementary Table 6. Mean growth measures and z-scores at different gestational ages 20](#_Toc189429295)

[Supplementary Table 6.1 Unadjusted mean growth measures and z-scores at different gestational ages by maternal HIV status. 20](#_Toc189429296)

[Supplementary Table 6.2 Adjusted mean growth measures and z-scores at different gestational ages by maternal HIV status. 22](#_Toc189429297)

[Supplementary Table 7. Mean growth velocity increments and z-scores at different gestational ages 24](#_Toc189429298)

[Supplementary Table 7.1 Unadjusted mean growth velocity increments and z-scores at different gestational ages by maternal HIV status. 24](#_Toc189429299)

[Supplementary Table 7.2 Adjusted mean growth velocity increments and z-scores at different gestational ages by maternal HIV status. 26](#_Toc189429300)

[Supplementary Table 8. Sensitivity analyses of LMM with highest quality ultrasound images 28](#_Toc189429301)

[Supplementary Table 9. Association of maternal HIV infection with in-utero SGA and VSGA at last antenatal ultrasound scan before delivery. 29](#_Toc189429302)

[Supplementary Table 10. Association of maternal HIV infection with SGA and VSGA at birth 30](#_Toc189429303)

[Supplementary Table 11. Adjusted mean growth measures and z-scores at different gestational ages for PWHIV who received EFV-based ART compared with HIV negative women. 31](#_Toc189429304)

[Supplementary Table 12. Linear mixed effects models specifications 35](#_Toc189429305)

[Supplementary Table 13. Outliers 36](#_Toc189429306)

# **Supplementary Figure 1. Directed acyclic graph of association of maternal HIV status with foetal growth.**


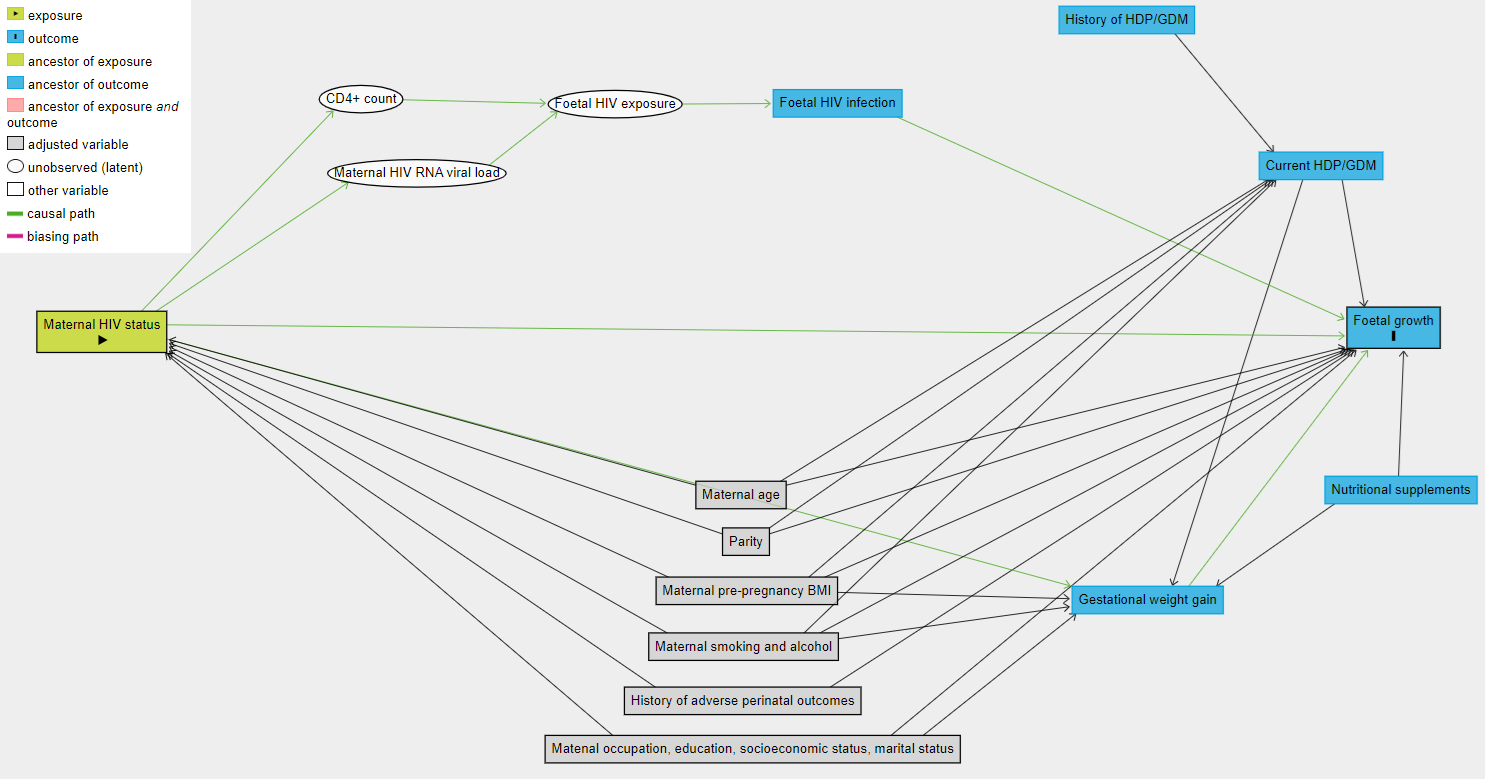


Abbreviations: HIV, Human immunodeficiency virus; CD4+, cluster determinant four positive; RNA, ribonucleic acid; HDP, hypertensive disorders in pregnancy; GDM, gestational diabetes mellitus; BMI, body mass index

# **Supplementary Figure 2. Flow diagram of study participants**

47 women excluded:

Loss to follow up (n=36)

Withdrawal of consent (n=11)

680 pregnant women enrolled

612 pregnant women included for analysis

**Measurements from 384 HIV-negative pregnant women:**

1. Head circumference: 1626

2. Biparietal diameter: 1627

3. Abdominal circumference: 1625

4. Femur length: 1626

**Measurements from 228 pregnant women living with HIV:**

1. Head circumference: 958
2. Biparietal diameter: 958
3. Abdominal circumference: 958
4. Femur length: 958

23 measurements excluded:

Outliers (< -5SD or > 5SD) (n=23)

21 excluded:

Missing biometry data (n=21)

633 pregnant women with data available for analyses

#

# **Supplementary Figure 3. Distribution of foetal growth velocity increments during pregnancy by maternal HIV status**


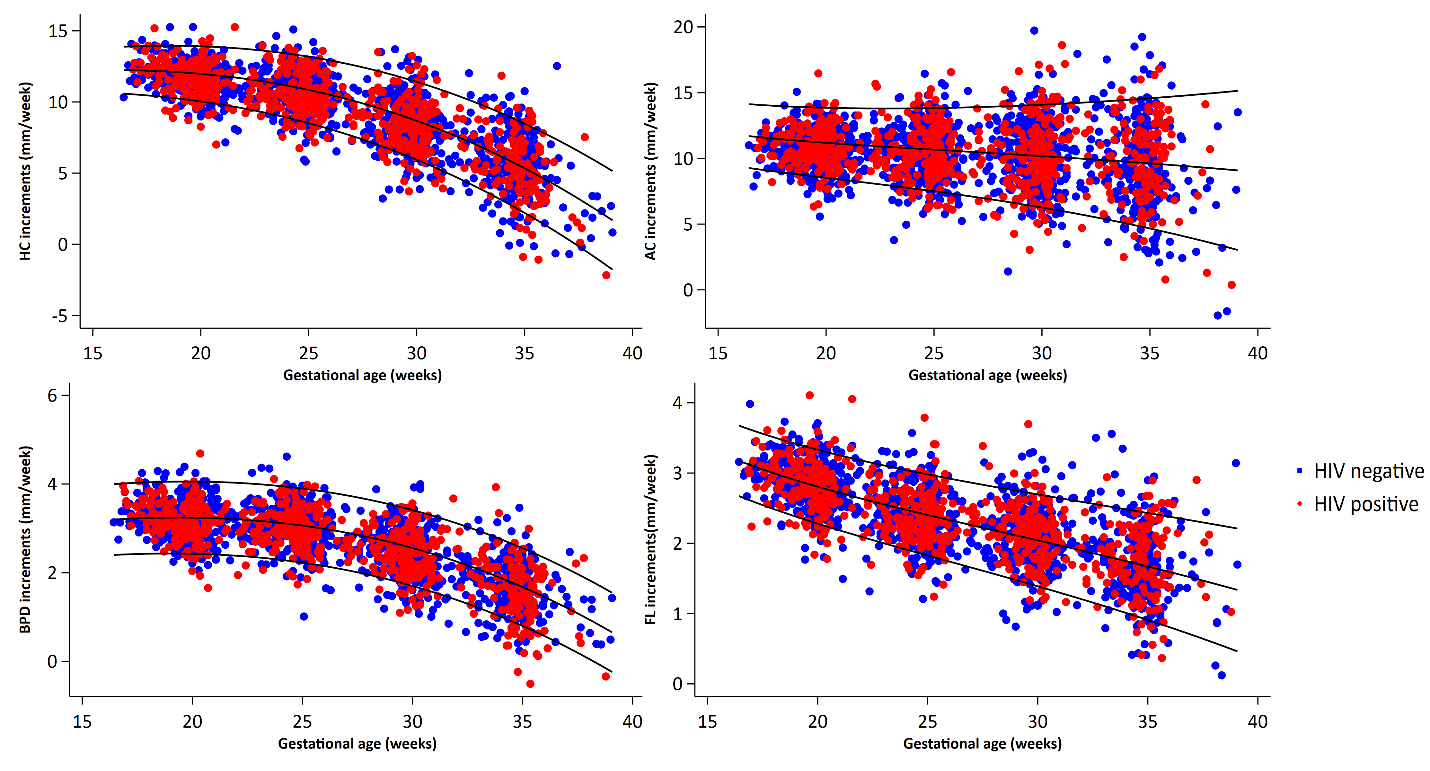


Dark lines representing 3^rd^ (bottom), 50^th^ (middle) and 97^th^ (top) centiles for respective foetal growth velocity increments from INTERGROWTH-21^st^ foetal growth standards.

Abbreviations: HIV, Human Immunodeficiency Virus; HC, head circumference; BPD, biparietal diameter; AC, abdominal circumference and FL, femur length.

**Supplementary Figure 4. Z-scores of mean foetal growth velocity increments by maternal HIV status**


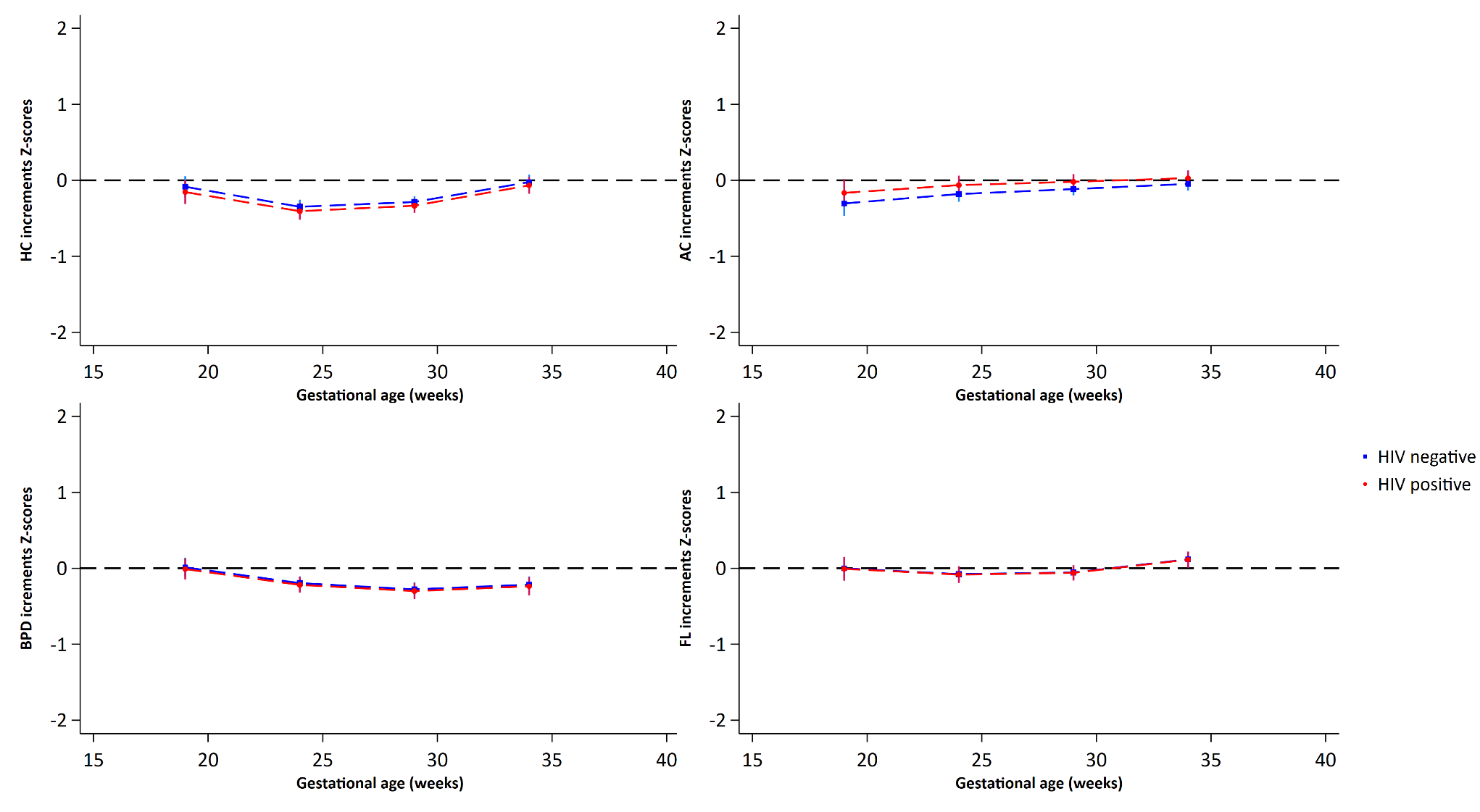


Z-scores of mean growth velocity increments measures calculated with reference to INTERGROWTH-21st foetal standards.

Means derived from multivariable linear mixed effects models adjusted for maternal age, baseline BMI, smoking, alcohol, parity, marital status, occupation, education, wealth index score, history of adverse perinatal outcomes and foetal sex.

Abbreviations: HIV, Human Immunodeficiency Virus; HC, head circumference; BPD, biparietal diameter; AC, abdominal circumference and FL, femur length.

**Supplementary Figure 5. Association of maternal HIV status with VSGA in-utero.**


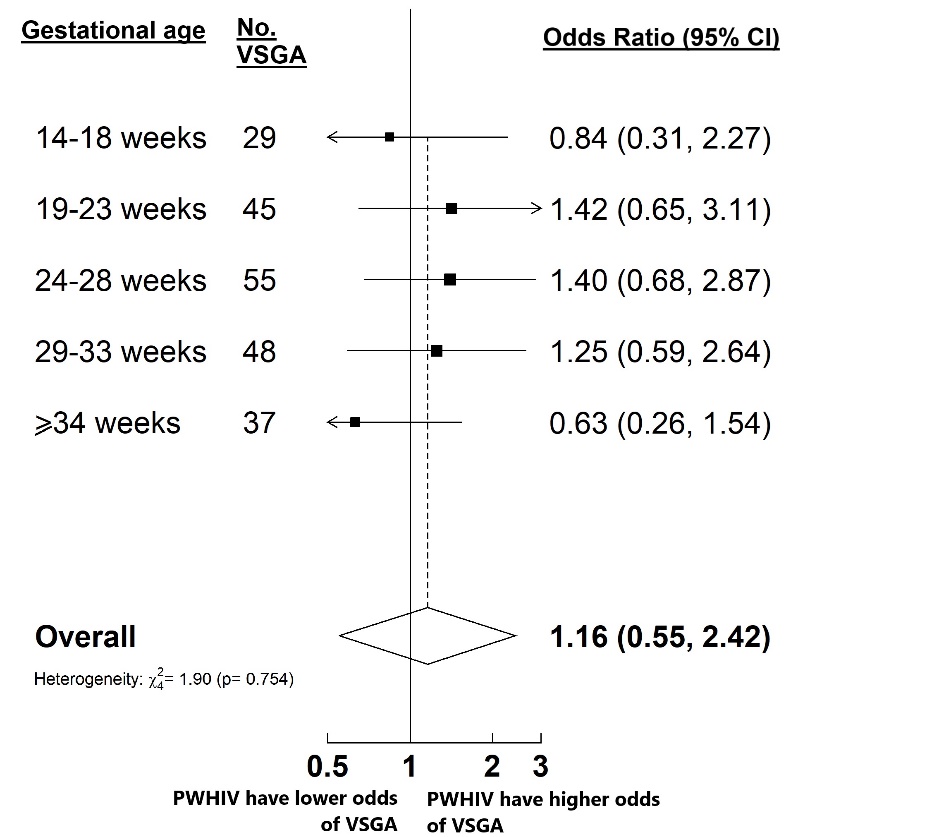


VSGA defined as estimated foetal weight (EFW) below 3^rd^ centile with reference to INTERGROWTH-21st foetal growth standards.

Adjusted for maternal age, baseline BMI, smoking, alcohol, parity, marital status, occupation, education, wealth index score, history of adverse perinatal outcomes, foetal sex and gestational age.

Abbreviations: VSGA, Very-small-for-gestational-age; OR, Odds Ratio; CI: confidence interval.

# **Supplementary Figure 6: Adjusted mean growth measures for PWHIV who received EFV-based ART compared with HIV negative women.**


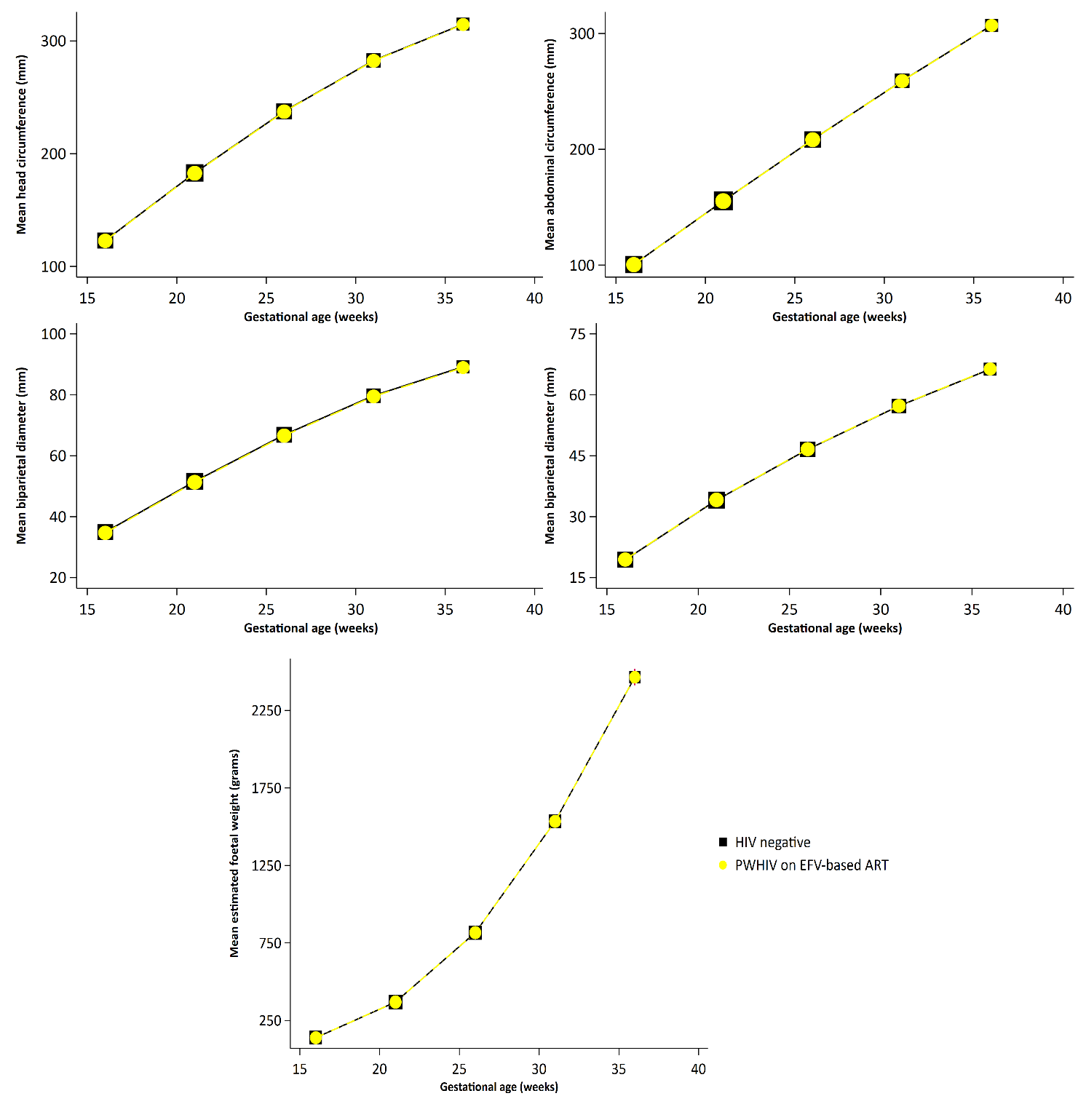


Means derived from multivariable linear mixed effects models adjusted for maternal age, baseline BMI, smoking, alcohol, parity, marital status, occupation, education, wealth index score, history of adverse perinatal outcomes and foetal sex.

Abbreviations: PWHIV, pregnant women living with HIV; ART, Antiretroviral therapy (triple drug therapy); HIV, Human Immunodeficiency Virus; EFV, Efavirenz (non-nucleoside reverse transcriptase inhibitor (NNRTI)).

**Supplementary Figure 7: Z-scores of adjusted mean growth measures for PWHIV who received EFV-based ART compared with HIV negative women.**


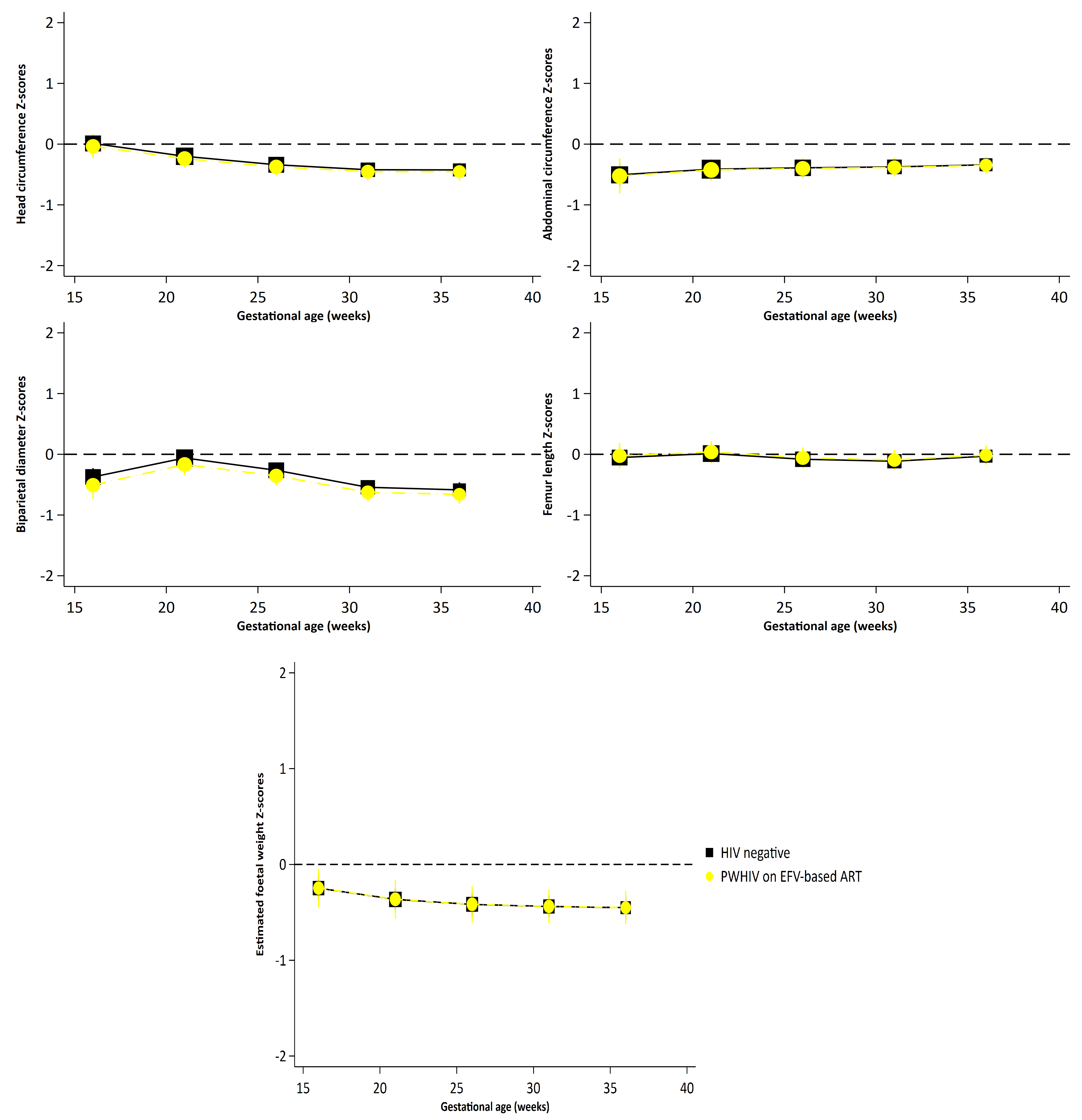


Z-scores of mean growth measures calculated with reference to INTERGROWTH-21^st^ foetal standards.

Means derived from multivariable linear mixed effects models adjusted for maternal age, baseline BMI, smoking, alcohol, parity, marital status, occupation, education, wealth index score, history of adverse perinatal outcomes and foetal sex.

Abbreviations: PWHIV, pregnant women living with HIV; ART, Antiretroviral therapy (triple drug therapy); HIV, Human Immunodeficiency Virus; EFV, Efavirenz (non-nucleoside reverse transcriptase inhibitor (NNRTI)).

| **Supplementary Table 1. Baseline characteristics of participants by HIV status.** | | | | |
| --- | --- | --- | --- | --- |
|  |  | **Maternal HIV status** | |  |
|  | **Total** | **HIV negative** | **HIV positive** | **p-value*** |
|  | **N=612** | **N=384** | **N=228** |  |
| Maternal age, mean (SD), years. | 30.6 (5.7) | 29.8 (5.7) | 31.9 (5.6) | <0.001 |
| BMI categories (WHO) (kg/m^2^) |  |  |  | 0.58 |
| Underweight (<18.5), n (%) | 11 (1.8) | 8 (2.1) | 3 (1.3) |  |
| Normal (18.5-24.9), n (%) | 210 (34.3) | 127 (33.1) | 83 (36.4) |  |
| Overweight (25-29.9), n (%) | 235 (38.4) | 154 (40.1) | 81 (35.5) |  |
| Obese (>30), n (%) | 156 (25.5) | 95 (24.7) | 61 (26.8) |  |
| Maternal smoking, n (%) | 45 (7.4) | 24 (6.3) | 21 (9.2) | 0.17 |
| Maternal alcohol, n (%) | 59 (9.6) | 37 (9.6) | 22 (9.6) | 1.00 |
| Parity |  |  |  | 0.007 |
| Nulliparous, n (%) | 100 (16.3) | 71 (18.4) | 29 (12.7) |  |
| Primiparous, n (%) | 264 (43.1) | 171 (44.5) | 93 (40.8) |  |
| Multiparous, n (%) | 211 (34.5) | 114 (29.7) | 97 (42.5) |  |
| Highest Education attained |  |  |  | 0.043 |
| No formal education, n (%) | 1 (0.2) | 0 (0.0) | 1 (0.4) |  |
| Primary education, n (%) | 19 (3.1) | 11 (2.9) | 8 (3.5) |  |
| Secondary education, n (%) | 452 (74.0) | 272 (71.0) | 180 (78.9) |  |
| Tertiary education, n (%) | 139 (22.7) | 100 (26.1) | 39 (17.1) |  |
| Married/Cohabiting, n (%) | 239 (39.1) | 158 (41.1) | 81 (35.5) | 0.17 |
| Occupation |  |  |  | 0.70 |
| Not working, n (%) | 79 (12.9) | 47 (12.2) | 32 (14.0) |  |
| Other, n (%) | 310 (50.7) | 193 (50.3) | 117 (51.3) |  |
| Working, n (%) | 223 (36.4) | 144 (37.5) | 79 (34.6) |  |
| Wealth Index Score ˟ |  |  |  | 0.100 |
| Low, n (%) | 208 (34.0) | 123 (32.0) | 85 (37.3) |  |
| Middle, n (%) | 200 (32.7) | 121 (31.5) | 79 (34.6) |  |
| High, n (%) | 204 (33.3) | 140 (36.5) | 64 (28.1) |  |
| History of adverse perinatal outcomes, n (%)§ | 184 (38.7) | 123 (43.2) | 61 (32.1) | 0.015 |
| History of low birthweight, n (%) | 94 (20.0) | 67 (23.7) | 27 (14.4) | 0.013 |
| History of preterm birth, n (%)† | 120 (25.3) | 76 (26.7) | 44 (23.2) | 0.39 |
| History of stillbirth, n (%)‡ | 60 (12.9) | 43 (15.1) | 17 (8.9) | 0.048 |
| History of neonatal death, n (%)° | 41 (8.6) | 23 (8.1) | 18 (9.5) | 0.59 |

Presenting mean (standard deviation, SD) for continuous variables and number (n) of observations (percentage) for categorical variable.

*Using independent two-sample t-test for continuous variables and Pearson’s chi-squared test for categorical variables.

§History of adverse perinatal outcomes was a composite variable for history of low birth weight, preterm birth, stillbirth and neonatal death.

˟Wealth Index Score generated from principal components analysis of asset-based measures at household level.

#Low birthweight defined as birthweight below 2500grams.

†Preterm birth defined as birth before 37 completed weeks of gestation.

‡Stillbirth defined as birth without any signs of life ≥ 24 weeks of gestation.

°Neonatal death defined as infant death in the first 28 days of life.

Abbreviations: HIV, Human Immunodeficiency Virus; BMI, Body Mass Index; WHO, World Health Organization.

# **Supplementary Table 2. Timing of HIV diagnosis**

|  | Total* | Preconception | Antenatal |
| --- | --- | --- | --- |
|  | N=106 |  |  |
| Timing of HIV diagnosis |  | 76 (71.7%) | 30 (28.3%) |
| Follow up visits at HIV diagnosis |  |  |  |
| During or before 1st antenatal visit, n (%) | |  | 29 (96.7%) |
| After 1st antenatal visit, n (%) | |  | 1 (3.3%) |
| Gestational age (weeks) at HIV diagnosis, median (IQR) | | | 8.0 (6.0-10.3) |
| Gestational age (weeks) at ART initiation, median (IQR) | | | 9.8 (7.0-11.0) |

* Includes all participants (n = 106) with information on dates of HIV diagnosis.

Preconception and antenatal defined with respect to estimated date of conception (EDC) determined by crown rump length.

# **Supplementary Table 3. Summary of ART use during pregnancy**

| **ART use in pregnancy** | | **Timing of ART initiation** | | |
| --- | --- | --- | --- | --- |
|  | **Total** | **Total** | **Preconception** | **Antenatal** |
|  | N=121 | N=110 | N=40 | N=70 |
| Antiretroviral drugs used in pregnancy |  |  |  |  |
| No, n (%) | 2 (1.7%) |  |  |  |
| Yes, n (%) | 119 (98.3%) | 110 (100.0%) | 40 (100.0%) | 70 (100.0%) |
| Type of antiretroviral drugs |  |  |  |  |
| Combination antiretroviral therapy, n (%) | 117 (98.3%) | 108 (98.2%) | 40 (100.0%) | 68 (97.1%) |
| Monotherapy, n (%) | 2 (1.7%) | 2 (1.8%) | 0 (0.0%) | 2 (2.9%) |
| Drug regimen |  |  |  |  |
| EFV-based, n (%) | 112 (94.1%) | 104 (94.5%) | 36 (90.0%) | 68 (97.1%) |
| NVP-based, n (%) | 2 (1.7%) | 2 (1.8%) | 2 (5.0%) | 0 (0.0%) |
| LPV/r (PI)-based, n (%) | 3 (2.5%) | 2 (1.8%) | 2 (5.0%) | 0 (0.0%) |
| AZT only (monotherapy), n (%) | 2 (1.7%) | 2 (1.8%) | 0 (0.0%) | 2 (2.9%) |

Information presented for 121 (out of 228) HIV-positive participants for whom ART use data was available.

Preconception initiation refers to starting ART use before conception of current pregnancy.

Antenatal initiation refers to starting ART use during the current pregnancy, after conception.

Abbreviations: ART, Antiretroviral therapy (triple drug therapy); AZT, Zidovudine (nucleoside reverse transcriptase inhibitor (NRTI)); EFV, Efavirenz (non-nucleoside reverse transcriptase inhibitor (NNRTI)); NVP, Nevirapine (NNRTI); LPV/r, Lopinavir/ritonavir; PI, Protease inhibitor; AZT, Zidovudine (nucleoside reverse transcriptase inhibitor (NRTI)).

# **Supplementary Table** **4. Foetal and newborn characteristics by maternal HIV status**

|  |  | **Maternal HIV status** | | |
| --- | --- | --- | --- | --- |
|  | **Total** | **HIV negative** | **HIV positive** | **p-value*** |
|  | N=612 | N=384 | N=228 |  |
| Gestational age at dating scan (by CRL), mean (SD), weeks. | 12.3 (11.0-13.1) | 12.3 (11.1-13.1) | 12.0 (11.0-13.3) | 0.62 |
| Number of follow up scans, median (IQR) | 5 (4.0-5.0) | 5 (4.0-5.0) | 5 (4.0-5.0) | 0.35 |
| Gestational age at last follow up scan, median (IQR) | 36.3 (32.6-37.4) | 36.4(32.6-37.4) | 36.1 (32.4-37.4) | 0.57 |
| Pregnancy outcome |  |  |  | 0.31 |
| Live birth, n (%) | 574 (93.8) | 356 (92.7) | 218 (95.6) |  |
| Stillbirth, n (%) | 16 (2.6) | 11 (2.9) | 5 (2.2) |  |
| Miscarriage, n (%) | 22 (3.6) | 17 (4.4) | 5 (2.2) |  |
| Total gestation at birth, mean (SD), weeks | 38.9 (37.4-39.9) | 38.9 (37.6-39.9) | 38.7 (37.1-39.9) | 0.43 |
| Mode of delivery |  |  |  | 0.89 |
| Caesarean section, n (%) | 339 (55.4) | 211 (54.9) | 128 (56.1) |  |
| Vaginal assisted, n (%) | 2 (0.3) | 1 (0.3) | 1 (0.4) |  |
| Vaginal spontaneous, n (%) | 271 (44.3) | 172 (44.8) | 99 (43.4) |  |
| Sex of baby |  |  |  | 0.80 |
| Female, n (%) | 287 (47.4) | 181 (47.8) | 106 (46.7) |  |
| Male, n (%) | 319 (52.6) | 198 (52.2) | 121 (53.3) |  |
| HIV status of baby (livebirths only) |  |  |  | 0.07 |
| HIV negative, n (%) | 572 (99.7) | 356 (100.0) | 216 (99.1) |  |
| Unknown, n (%) | 2 (0.3) | 0(0.0) | 2 (0.9) |  |
| Weight at delivery, mean (SD), grams | 2990.0 (2595.0-3260.0) | 2995.0 (2650.0-3265.0) | 2962.5 (2540.0-3250.0) | 0.22 |
| Birthweight Z-score, median (IQR)** | -0.4 (-1.1-0.3) | -0.4 (-1.0-0.4) | -0.5 (-1.2-0.3) | 0.34 |
| Birthweight centile, median (IQR)** | 33.0 (13.2-62.9) | 34.0 (14.9-63.8) | 32.0 (11.3-61.2) | 0.34 |
| Preterm birth, n (%) | 99 (17.2) | 55 (15.4) | 44 (20.2) | 0.15 |
| Low birthweight, n (%) | 115 (20.3) | 66 (18.7) | 49 (22.9) | 0.23 |
| Very low birthweight, n (%) | 22 (3.9) | 14 (4.0) | 8 (3.7) | 0.89 |
| Small for gestational age, n (%) | 107 (18.9) | 58 (16.5) | 49 (23.0) | 0.06 |
| Very small for gestational age, n (%) | 30 (5.3) | 16 (4.5) | 14 (6.6) | 0.30 |

Presenting median (interquartile range) or mean (standard deviation) for continuous variables and number of observations (percentage) for categorical variables.

*Using independent two-sample t-test or Wilcoxon rank-sum test, as appropriate, for continuous variables and Pearson’s chi-square test for categorical variables.

** Calculated in reference to INTERGROWTH-21st standards for newborns.

Preterm birth defined as livebirth before 37 completed weeks of gestation.

Low birth weight defined as birthweight < 2500grams.

Very low birth weight defined as birthweight < 1500grams. Small for gestational age defined as birthweight < 10^th^ centile.

Small for gestational age defined as birthweight < 10^th^ centile.

Very small for gestational age defined as birthweight < 3^rd^ centile.

Abbreviations: HIV, Human Immunodeficiency Virus; CRL, Crown Rump Length;

# **Supplementary Table 5. Summary of antenatal follow up and unadjusted foetal growth z-scores and prevalence of SGA and VSGA.**

|  | **Total** | **HIV negative** | **HIV positive** | **p-value*** |
| --- | --- | --- | --- | --- |
| **14-18 weeks** | **N=554** | **N=349** | **N=205** |  |
| Abdominal circumference˟ | -0.47 (-1.11, 0.26) | -0.54 (-1.17, 0.24) | -0.44 (-1.00, 0.29) | 0.24 |
| Biparietal diameter˟ | -0.17 (-0.82, 0.45) | -0.17 (-0.82, 0.44) | -0.20 (-0.82, 0.49) | 0.65 |
| Head circumference˟ | -0.08 (-0.64, 0.52) | -0.09 (-0.59, 0.46) | -0.03 (-0.68, 0.54) | 0.84 |
| Femur length˟ | -0.14 (-0.65, 0.54) | -0.19 (-0.66, 0.51) | -0.08 (-0.62, 0.63) | 0.18 |
| Estimated foetal weight˟ | -0.27 (-1.04, 0.33) | -0.38 (-1.11, 0.29) | -0.21 (-0.91, 0.35) | 0.18 |
| SGA**^†^** | 94 (17.0) | 65 (18.6) | 29 (14.2) | 0.18 |
| VSGA**^†^** | 29 (5.2) | 20 (5.7) | 9 (4.4) | 0.49 |
| **19-23 weeks** | **N=571** | **N=358** | **N=213** |  |
| Abdominal circumference˟ | -0.38 (-1.07, 0.24) | -0.38 (-1.14, 0.20) | -0.34 (-0.98, 0.25) | 0.33 |
| Biparietal diameter˟ | -0.19 (-0.86, 0.43) | -0.16 (-0.79, 0.43) | -0.26 (-0.93, 0.42) | 0.36 |
| Head circumference˟ | -0.31 (-0.85, 0.28) | -0.30 (-0.82, 0.22) | -0.31 (-0.91, 0.37) | 0.80 |
| Femur length˟ | 0.02 (-0.66, 0.70) | -0.01 (-0.67, 0.70) | 0.12 (-0.60, 0.74) | 0.57 |
| Estimated foetal weight˟ | -0.37 (-1.08, 0.32) | -0.39 (-1.08, 0.31) | -0.34 (-1.08, 0.33) | 0.48 |
| SGA**^†^** | 103 (18.1) | 66 (18.5) | 37 (17.4) | 0.74 |
| VSGA**^†^** | 45 (7.9) | 26 (7.3) | 19 (8.9) | 0.48 |
| **24-28 weeks** | **N=537** | **N=336** | **N=201** |  |
| Abdominal circumference˟ | -0.42 (-1.12, 0.31) | -0.39 (-1.10, 0.21) | -0.60 (-1.14, 0.50) | 0.81 |
| Biparietal diameter˟ | -0.35 (-1.01, 0.38) | -0.31 (-0.99, 0.47) | -0.42 (-1.03, 0.31) | 0.35 |
| Head circumference˟ | -0.41 (-1.02, 0.26) | -0.34 (-1.01, 0.26) | -0.49 (-1.10, 0.25) | 0.31 |
| Femur length˟ | -0.15 (-0.84, 0.56) | -0.18 (-0.84, 0.58) | -0.11 (-0.84, 0.49) | 0.71 |
| Estimated foetal weight˟ | -0.46 (-1.24, 0.21) | -0.45 (-1.18, 0.20) | -0.48 (-1.35, 0.24) | 0.81 |
| SGA**^†^** | 128 (23.9) | 75 (22.4) | 53 (26.4) | 0.30 |
| VSGA**^†^** | 55 (10.3) | 31 (9.3) | 24 (11.9) | 0.32 |
| **29-33 weeks** | **N=498** | **N=312** | **N=186** |  |
| Abdominal circumference˟ | -0.41 (-1.13, 0.47) | -0.33 (-1.10, 0.45) | -0.49 (-1.19, 0.50) | 0.78 |
| Biparietal diameter˟ | -0.54 (-1.37, 0.21) | -0.50 (-1.41, 0.29) | -0.69 (-1.29, 0.02) | 0.21 |
| Head circumference˟ | -0.49 (-1.26, 0.27) | -0.47 (-1.21, 0.31) | -0.57 (-1.41, 0.22) | 0.28 |
| Femur length˟ | -0.07 (-0.78, 0.71) | -0.10 (-0.81, 0.69) | 0.00 (-0.78,0.79) | 0.80 |
| Estimated foetal weight˟ | -0.48 (-1.27, 0.32) | -0.48 (-1.27, 0.34) | -0.47 (-1.27, 0.29) | 0.82 |
| SGA**^†^** | 120 (24.1) | 75 (24.1) | 45 (24.2) | 0.98 |
| VSGA**^†^** | 48 (9.7) | 30 (9.7) | 18 (9.7) | 0.99 |
| **34-38 weeks** | **N=405** | **N=256** | **N=149** |  |
| Abdominal circumference˟ | -0.33 (-0.97, 0.43) | -0.40 (-1.02, 0.47) | -0.26 (-0.90, 0.38) | 0.33 |
| Biparietal diameter˟ | -0.59 (-1.40, 0.15) | -0.65 (-1.35, 0.20) | -0.57 (-1.62, 0.10) | 0.36 |
| Head circumference˟ | -0.55 (-1.27, 0.26) | -0.52 (-1.22, 0.28) | -0.65 (-1.36, 0.16) | 0.34 |
| Femur length˟ | 0.03 (-0.76, 0.91) | 0.05 (-0.75, 0.85) | -0.01 (-0.78, 0.93) | 0.84 |
| Estimated foetal weight˟ | -0.43 (-1.17, 0.27) | -0.44 (-1.18, 0.27) | -0.42 (-1.16, 0.37) | 0.55 |
| SGA**^†^** | 82 (20.2) | 55 (21.4) | 27 (18.1) | 0.43 |
| VSGA**^†^** | 32 (7.9) | 23 (9.0) | 9 (6.0) | 0.29 |
| **≥ 39 weeks** | **N=19** | **N=15** | **N=4** |  |
| Abdominal circumference˟ | -1.12 (-1.48, -0.13) | -1.11 (-1.65, -0.26) | -1.12 (-1.20, 0.84) | 0.41 |
| Biparietal diameter˟ | -0.70 (-2.08, -0.36) | -1.14 (-2.14, -0.39) | -0.70 (-1.17, 0.50) | 0.54 |
| Head circumference˟ | -0.59 (-2.36, -0.07) | -1.35 (-2.40, -0.15) | -0.31 (-0.59, 1.07) | 0.22 |
| Femur length˟ | 0.71 (-0.87, -1.26) | 0.37 (-1.26, 1.06) | 0.83 (0.52, 1.74) | 0.41 |
| Estimated foetal weight˟ | -0.84 (-2.01, -0.05) | -1.14 (-2.11, -0.05) | -0.72 (-1.03, 0.29) | 0.48 |
| SGA**^†^** | 7 (33.3) | 7 (41.2) | 0 (0.0) | 0.12 |
| VSGA**^†^** | 5 (23.8) | 5 (29.4) | 0 (0.0) | 0.21 |

˟Median (interquartile range). ^†^Number of cases (%).

* p-value of Wilcoxon rank-sum test for continuous variables and Pearson's chi-square test between the two categories for SGA and VSGA.

Gestational ages represent completed weeks.

Z-scores calculated with reference to INTERGROWTH-21st foetal growth standards.

SGA (Estimated foetal weight (EFW) < 10^th^ centile) and VSGA (EFW < 3^rd^ centile) defined with reference to INTERGROWTH-21^st^ standards for foetal growth.

Abbreviations: SGA, Small-for-gestational-age; VSGA, Very-small-for-gestational-age.

# **Supplementary Table 6. Mean growth measures and z-scores at different gestational ages**

## **Supplementary Table 6.1 Unadjusted mean growth measures and z-scores at different gestational ages by maternal HIV status.**

|  | **16 weeks** | | **21 weeks** | **26 weeks** | | **31 weeks** | **36 weeks** | **Overall difference by HIV status** | **p-value*** |
| --- | --- | --- | --- | --- | --- | --- | --- | --- | --- |
| **Head circumference** | | | | |  |  |  |  |  |
| **HIV negative** | |  | | |  |  |  |  |  |
| Mean (mm) | 122.78 (122.08, 123.48) | 182.85 (182.24, 183.46) | | | 237.38 (236.64, 238.11) | 282.18 (281.30, 283.06) | 313.82 (312.72, 314.92) |  |  |
| Mean (Z-score) | -0.02 (0.09, -0.13) | -0.22 (-0.14, -0.30) | | | -0.36 (-0.28, -0.45) | -0.47 (-0.38, -0.57) | -0.52 (-0.42, -0.62) |  |  |
| **HIV positive** | |  | | |  |  |  |  |  |
| Mean (mm) | 122.62 (121.76, 123.48) | 182.69 (181.90, 183.48) | | | 237.22 (236.33, 238.10) | 282.02 (281.01, 283.03) | 313.66 (312.46, 314.86) |  |  |
| Mean (Z-score) | -0.05 (0.09, -0.18) | -0.24 (-0.13, -0.34) | | | -0.38 (-0.28, -0.48) | -0.49 (-0.38, -0.60) | -0.53 (-0.42, -0.65) |  |  |
| **Mean difference˟** | **-0.16 (-1.27, 0.95)** | **-0.16 (-0.91, 1.09)** | | | **-0.16 (-1.31, 0.99)** | **-0.16 (-1.50, 1.18)** | **-0.16 (-1.79, 1.47)** | **-0.16 (-1.15, 0.82)** | **0.75** |
| **Biparietal diameter** | | | | |  |  |  |  |  |
| **HIV negative** | |  | | |  |  |  |  |  |
| Mean (mm) | 34.99 (34.75, 35.23) | 51.59 (51.38, 51.80) | | | 66.84 (66.60, 67.09) | 79.62 (79.32, 79.91) | 88.99 (88.63, 89.36) |  |  |
| Mean (Z-score) | -0.34 (-0.22, -0.47) | -0.05 (0.04, -0.13) | | | -0.27 (-0.18, -0.36) | -0.58 (-0.48, -0.67) | -0.65 (-0.54, -0.75) |  |  |
| **HIV positive** | |  | | |  |  |  |  |  |
| Mean (mm) | 34.90 (34.61, 35.19) | 51.50 (51.23, 51.77) | | | 66.75 (66.45, 67.05) | 79.53 (79.19, 79.86) | 88.90 (88.50, 89.30) |  |  |
| Mean (Z-score) | -0.39 (-0.24, -0.54) | -0.09 (0.03, -0.20) | | | -0.30 (-0.20, -0.41) | -0.61 (-0.50, -0.71) | -0.67 (-0.56, -0.79) |  |  |
| **Mean difference˟** | **-0.09 (-0.47, 0.29)** | **-0.09 (-0.31, 0.37)** | | | **-0.09 (-0.47, 0.30)** | **-0.09 (-0.53, 0.36)** | **-0.09 (-0.63, 0.45)** | **-0.09 (-0.43, 0.25)** | **0.60** |
| **Abdominal circumference** | | | | | |  |  |  |  |
| **HIV negative** | |  | | |  |  |  |  |  |
| Mean (mm) | 100.50 (99.61, 101.38) | 155.15 (154.41, 155.89) | | | 208.06 (207.09, 209.04) | 258.83 (257.60, 260.06) | 307.03 (305.43, 308.63) |  |  |
| Mean (Z-score) | -0.50 (-0.34, -0.66) | -0.44 (-0.35, -0.53) | | | -0.42 (-0.33, -0.51) | -0.39 (-0.30, -0.49) | -0.34 (-0.25, -0.43) |  |  |
| **HIV positive** | |  | | |  |  |  |  |  |
| Mean (mm) | 100.73 (99.67, 101.79) | 155.38 (154.44, 156.33) | | | 208.30 (207.16, 209.44) | 259.07 (257.70, 260.43) | 307.27 (305.57, 308.96) |  |  |
| Mean (Z-score) | -0.46 (-0.26, -0.65) | -0.41 (-0.29, -0.52) | | | -0.40 (-0.29, -0.51) | -0.38 (-0.27, -0.48) | -0.33 (-0.23, -0.43) |  |  |
| **Mean difference˟** | **0.24 (-1.14, 1.61)** | **0.24 (-1.09, 1.30)** | | | **0.24 (-1.26, 1.73)** | **0.24 (-1.60, 2.07)** | **0.24 (-2.09, 2.57)** | **0.13 (-1.09, 1.36)** | **0.83** |
| **Femur length** | |  | | |  |  |  |  |  |
| **HIV negative** | |  | | |  |  |  |  |  |
| Mean (mm) | 19.37 (19.18, 19.56) | 34.05 (33.88, 34.22) | | | 46.54 (46.34, 46.73) | 57.21 (56.98, 57.44) | 66.26 (65.98, 66.54) |  |  |
| Mean (Z-score) | -0.07 (0.05, -0.18) | 0.00 (0.09, -0.10) | | | -0.10 (0.00, -0.20) | -0.13 (-0.03, -0.23) | -0.05 (0.05, -0.15) |  |  |
| **HIV positive** | |  | | |  |  |  |  |  |
| Mean (mm) | 19.39 (19.16, 19.62) | 34.07 (33.86, 34.29) | | | 46.56 (46.32, 46.80) | 57.24 (56.97, 57.50) | 66.29 (65.98, 66.60) |  |  |
| Mean (Z-score) | -0.05 (0.09, -0.19) | 0.01 (0.13, -0.11) | | | -0.09 (0.03, -0.20) | -0.12 (-0.01, -0.24) | -0.04 (0.07, -0.15) |  |  |
| **Mean difference˟** | **0.02 (-0.27, 0.32)** | **0.02 (-0.25, 0.30)** | | | **0.02 (-0.28, 0.33)** | **0.02 (-0.32, 0.37)** | **0.02 (-0.39, 0.44)** | **0.02 (-0.24, 0.29)** | **0.86** |
| **Estimated foetal weight** | | | | | |  |  |  |  |
| **HIV negative** | |  | | |  |  |  |  |  |
| Mean (grams) | 138.78 (137.36, 140.21) | 368.04 (364.70, 371.41) | | | 813.43 (805.45, 821.50) | 1528.06 (1511.53, 1544.78) | 2455.12 (2423.70, 2486.96) |  |  |
| Mean (Z-score) | -0.27 (-0.38, -0.17) | -0.41 (-0.52, -0.31) | | | -0.47 (-0.58, -0.37) | -0.49 (-0.59, -0.39) | -0.50 (-0.60, -0.39) |  |  |
| **HIV positive** | |  | | |  |  |  |  |  |
| Mean (grams) | 139.20 (137.44, 140.99) | 369.16 (364.83, 373.54) | | | 815.90 (805.87, 826.06) | 1532.70 (1512.62, 1553.05) | 2462.58 (2426.18, 2499.51) |  |  |
| Mean (Z-score) | -0.24 (-0.38, -0.11) | -0.38 (-0.51, -0.25) | | | -0.44 (-0.57, -0.32) | -0.47 (-0.59, -0.35) | -0.47 (-0.60, -0.35) |  |  |
| **Mean difference˟** | **0.42 (-2.37, 3.21)** | **1.12 (-1.67, 3.90)** | | | **2.46 (-0.32, 5.25)** | **4.63 (1.84, 7.42)** | **7.44 (4.65, 10.23)** | **1.00 (0.99, 1.02)** | **0.69** |
|  | | | | | | | | | |

Presented as predicted mean (95% confidence interval).

* p-value for overall difference in mean growth measures between HIV-positive mothers and HIV-negative mothers.

˟ mean difference in growth measures between HIV-positive mothers and HIV-negative mothers at specified gestational ages.

Z-scores calculated with reference to INTERGROWTH-21^st^ foetal growth standards.

## **Supplementary Table 6.2 Adjusted mean growth measures and z-scores at different gestational ages by maternal HIV status.**

|  | **16 weeks** | **21 weeks** | **26 weeks** | **31 weeks** | **36 weeks** | **Overall difference by HIV status** | **p*** | **p_int_**** |
| --- | --- | --- | --- | --- | --- | --- | --- | --- |
| **Head circumference** | |  |  |  |  |  |  |  |
| **HIV negative** | |  |  |  |  |  |  |  |
| Mean (mm) | 122.89 (122.10, 123.69) | 182.88 (182.20, 183.57) | 237.49 (236.68, 238.30) | 282.57 (281.59, 283.54) | 314.69 (313.47, 315.91) |  |  |  |
| Mean (Z-score) | 0.00 (-0.13, 0.12) | -0.21 (-0.30, -0.12) | -0.35 (-0.44, -0.26) | -0.43 (-0.54, -0.33) | -0.44 (-0.55, -0.32) |  |  |  |
| **HIV positive** | |  |  |  |  |  |  |  |
| Mean (mm) | 122.53 (121.60, 123.46) | 182.52 (181.68, 183.35) | 237.12 (236.18, 238.07) | 282.20 (281.11, 283.28) | 314.33 (313.02, 315.63) |  |  |  |
| Mean (Z-score) | -0.06 (-0.21, 0.09) | -0.26 (-0.37, -0.15) | -0.39 (-0.50, -0.28) | -0.47 (-0.59, -0.36) | -0.47 (-0.59, -0.35) |  |  |  |
| **Mean difference˟** | **-0.37 (-1.60, 0.86)** | **-0.36 (-1.00, 1.16)** | **-0.37 (-1.61, 0.88)** | **-0.37 (-1.82, 1.09)** | **-0.37 (-2.16, 1.42)** | **-0.37 (-1.46, 0.72)** | **0.51** | **0.25** |
| **Biparietal diameter** | |  |  |  |  |  |  |  |
| **HIV negative** | |  |  |  |  |  |  |  |
| Mean (mm) | 34.93 (34.66 - 35.20) | 51.52 (51.29 - 51.76) | 66.80 (66.53 - 67.08) | 79.65 (79.33 - 79.98) | 89.15 (88.74 - 89.55) |  |  |  |
| Mean (Z-score) | -0.37 (-0.52, -0.23) | -0.08 (-0.17, 0.02) | -0.29 (-0.38, -0.19) | -0.57 (-0.67, -0.46) | -0.60 (-0.72, -0.49) |  |  |  |
| **HIV positive** | |  |  |  |  |  |  |  |
| Mean (mm) | 34.86 (34.54 - 35.18) | 51.45 (51.17 - 51.74) | 66.73 (66.41 - 67.05) | 79.58 (79.22 - 79.94) | 89.08 (88.64 - 89.51) |  |  |  |
| Mean (Z-score) | -0.41 (-0.58, -0.25) | -0.11 (-0.23, 0.01) | -0.31 (-0.42, -0.20) | -0.59 (-0.70, -0.47) | -0.62 (-0.75, -0.50) |  |  |  |
| **Mean difference˟** | **-0.07 (-0.49, 0.35)** | **-0.07 (-0.34, 0.40)** | **-0.07 (-0.49, 0.35)** | **-0.07 (-0.56, 0.42)** | **-0.07 (-0.67, 0.52)** | **-0.07 (-0.45, 0.30)** | **0.70** | **0.26** |
| **Abdominal circumference** | | |  |  |  |  |  |  |
| **HIV negative** | |  |  |  |  |  |  |  |
| Mean (mm) | 100.67 (99.66, 101.69) | 155.42 (154.58, 156.27) | 208.53 (207.42, 209.64) | 259.60 (258.20, 261.01) | 308.25 (306.42, 310.08) |  |  |  |
| Mean (Z-score) | -0.47 (-0.66, -0.28) | -0.40 (-0.51, -0.30) | -0.37 (-0.48, -0.27) | -0.33 (-0.44, -0.23) | -0.27 (-0.38, -0.16) |  |  |  |
| **HIV positive** | |  |  |  |  |  |  |  |
| Mean (mm) | 100.54 (99.38, 101.71) | 155.29 (154.27, 156.32) | 208.40 (207.14, 209.66) | 259.47 (257.95, 260.99) | 308.12 (306.20, 310.03) |  |  |  |
| Mean (Z-score) | -0.49 (-0.71, -0.28) | -0.42 (-0.55, -0.29) | -0.39 (-0.51, -0.27) | -0.34 (-0.46, -0.23) | -0.28 (-0.39, -0.16) |  |  |  |
| **Mean difference˟** | **-0.13 (-1.68, 1.41)** | **-0.13 (-1.24, 1.42)** | **-0.13 (-1.81, 1.55)** | **-0.13 (-2.20, 1.94)** | **-0.13 (-2.78, 2.52)** | **-0.15 (-1.47, 1.17)** | **0.83** | **0.58** |
| **Femur length** | |  |  |  |  |  |  |  |
| **HIV negative** | |  |  |  |  |  |  |  |
| Mean (mm) | 19.38 (19.16, 19.59) | 34.06 (33.87, 34.25) | 46.55 (46.33, 46.77) | 57.23 (56.97, 57.48) | 66.28 (65.97, 66.60) |  |  |  |
| Mean (Z-score) | -0.06 (-0.19, 0.07) | 0.00 (-0.10, 0.11) | -0.09 (-0.20, 0.02) | -0.13 (-0.24, -0.02) | -0.04 (-0.16, 0.07) |  |  |  |
| **HIV positive** | |  |  |  |  |  |  |  |
| Mean (mm) | 19.41 (19.16, 19.66) | 34.09 (33.86, 34.33) | 46.58 (46.32, 46.84) | 57.26 (56.97, 57.55) | 66.32 (65.98, 66.66) |  |  |  |
| Mean (Z-score) | -0.04 (-0.19, 0.12) | 0.02 (-0.11, 0.15) | -0.08 (-0.20, 0.05) | -0.11 (-0.24, 0.01) | -0.03 (-0.15, 0.10) |  |  |  |
| **Mean difference˟** | **0.04 (-0.29, 0.37)** | **0.03 (-0.28, 0.32)** | **0.04 (-0.31, 0.38)** | **0.04 (-0.35, 0.42)** | **0.04 (-0.43, 0.50)** | **0.04 (-0.27, 0.34)** | **0.82** | **0.53** |
| **Estimated foetal weight** | | |  |  |  |  |  |  |
| **HIV negative** | |  |  |  |  |  |  |  |
| Mean (grams) | 138.91 (137.25, 140.59) | 368.81 (364.93, 372.73) | 816.40 (807.20, 825.69) | 1536.78 (1517.75, 1556.04) | 2475.75 (2439.47, 2512.56) |  |  |  |
| Mean (Z-score) | -0.26 (-0.39, -0.14) | -0.39 (-0.51, -0.27) | -0.44 (-0.55, -0.32) | -0.44 (-0.56, -0.33) | -0.43 (-0.55, -0.31) |  |  |  |
| **HIV positive** | |  |  |  |  |  |  |  |
| Mean (grams) | 139.00 (137.05, 140.98) | 369.05 (364.30, 373.86) | 816.93 (805.92, 828.09) | 1537.78 (1515.61, 1560.28) | 2477.37 (2436.73, 2518.68) |  |  |  |
| Mean (Z-score) | -0.26 (-0.41, -0.11) | -0.38 (-0.53, -0.24) | -0.43 (-0.57, -0.29) | -0.44 (-0.57, -0.30) | -0.42 (-0.56, -0.29) |  |  |  |
| **Mean difference˟** | **0.09 (-2.70, 2.88)** | **0.24 (-2.55, 3.03)** | **0.53 (-2.26, 3.32)** | **1.01 (-1.79, 3.80)** | **1.62 (-1.17, 4.41)** | **1.00 (0.98, 1.02)** | **0.95** | **0.49** |

Presented as predicted mean (95% confidence interval).

Estimates adjusted for maternal age, baseline BMI, smoking, alcohol, parity, marital status, occupation, education, wealth index score, history of adverse perinatal outcomes and foetal sex.

* p-value for overall difference in mean growth measures between HIV-positive mothers and HIV-negative mothers.

˟ mean difference in growth measures between HIV-positive mothers and HIV-negative mothers at specified gestational ages.

Z-scores calculated with reference to INTERGROWTH-21st foetal growth standards.

**Supplementary Table 7. Mean growth velocity increments and z-scores at different gestational ages**

**Supplementary Table 7.1 Unadjusted mean growth velocity increments and z-scores at different gestational ages by maternal HIV status.**

|  | **19 weeks** | **24 weeks** | **29 weeks** | **34 weeks** | **Overall difference by HIV status** | **p*** |
| --- | --- | --- | --- | --- | --- | --- |
| **Head circumference increments** | | | | |  |  |
| **HIV negative** | |  |  |  |  |  |
| Mean (mm/week) | 11.98 (11.87, 12.10) | 10.69 (10.60, 10.79) | 8.74 (8.65, 8.83) | 5.98 (5.83, 6.13) |  |  |
| Mean (Z-score) | -0.11 (-0.23, 0.01) | -0.38 (-0.46, -0.30) | -0.33 (-0.39, -0.26) | -0.08 (-0.17, 0.01) |  |  |
| **HIV positive** | |  |  |  |  |  |
| Mean (mm/week) | 11.94 (11.80, 12.08) | 10.65 (10.53, 10.78) | 8.70 (8.58, 8.82) | 5.94 (5.77, 6.10) |  |  |
| Mean (Z-score) | -0.15 (-0.29, -0.01) | -0.41 (-0.51, -0.31) | -0.36 (-0.44, -0.27) | -0.10 (-0.21, 0.00) |  |  |
| **Mean difference˟** | **-0.04 (-0.22, 0.14)** | **-0.04 (-0.20, 0.11)** | **-0.04 (-0.19, 0.11)** | **-0.04 (-0.26, 0.18)** | **-0.04 (-0.19, 0.11)** | **0.58** |
| **Biparietal diameter increments** | | | | |  |  |
| **HIV negative** | |  |  |  |  |  |
| Mean (mm/week) | 3.24 (3.19 - 3.28) | 3.03 (2.99 - 3.06) | 2.55 (2.51 - 2.59) | 1.76 (1.72 - 1.80) |  |  |
| Mean (Z-score) | -0.01 (-0.12 - 0.10) | -0.21 (-0.29 - -0.14) | -0.31 (-0.39 - -0.22) | -0.27 (-0.37 - -0.18) |  |  |
| **HIV positive** | |  |  |  |  |  |
| Mean (mm/week) | 3.23 (3.18 - 3.28) | 3.02 (2.98 - 3.06) | 2.54 (2.50 - 2.58) | 1.75 (1.70 - 1.80) |  |  |
| Mean (Z-score) | -0.03 (-0.15 - 0.09) | -0.23 (-0.33 - -0.14) | -0.33 (-0.42 - -0.23) | -0.29 (-0.40 - -0.19) |  |  |
| **Mean difference˟** | **-0.01 (-0.08, 0.06)** | **-0.01 (-0.06, 0.04)** | **-0.01 (-0.07, 0.05)** | **-0.01 (-0.07, 0.06)** | **-0.01 (-0.05, 0.04)** | **0.70** |
| **Abdominal circumference** | | | |  |  |  |
| **HIV negative** | |  |  |  |  |  |
| Mean (mm/week) | 10.88 (10.68 - 11.07) | 10.44 (10.30 - 10.58) | 10.01 (9.87 - 10.15) | 9.57 (9.38 - 9.77) |  |  |
| Mean (Z-score) | -0.31 (-0.45 - -0.17) | -0.20 (-0.28 - -0.11) | -0.13 (-0.20 - -0.06) | -0.07 (-0.14 - 0.01) |  |  |
| **HIV positive** | |  |  |  |  |  |
| Mean (mm/week) | 11.03 (10.81 - 11.25) | 10.59 (10.42 - 10.77) | 10.16 (9.98 - 10.34) | 9.72 (9.50 - 9.95) |  |  |
| Mean (Z-score) | -0.20 (-0.37 - -0.04) | -0.10 (-0.21 - 0.01) | -0.06 (-0.15 - 0.03) | -0.01 (-0.10 - 0.08) |  |  |
| **Mean difference˟** | **0.15 (-0.15, 0.45)** | **0.15 (-0.08, 0.38)** | **0.15 (-0.08, 0.38)** | **0.15 (-0.15, 0.45)** | **0.15 (-0.07, 0.37)** | **0.17** |
| **Femur length** | | |  |  |  |  |
| **HIV negative** | |  |  |  |  |  |
| Mean (mm/week) | 2.89 (2.86, 2.93) | 2.45 (2.43, 2.47) | 2.09 (2.07, 2.12) | 1.79 (1.76, 1.82) |  |  |
| Mean (Z-score) | -0.02 (-0.14, 0.11) | -0.09 (-0.17, -0.01) | -0.07 (-0.14, 0.01) | 0.11 (0.03, 0.19) |  |  |
| **HIV positive** | |  |  |  |  |  |
| Mean (mm/week) | 2.90 (2.86, 2.94) | 2.46 (2.43, 2.49) | 2.10 (2.07, 2.13) | 1.80 (1.76, 1.83) |  |  |
| Mean (Z-score) | 0.01 (-0.14, 0.15) | -0.07 (-0.17, 0.03) | -0.05 (-0.14, 0.04) | 0.12 (0.03, 0.22) |  |  |
| **Mean difference˟** | **0.01 (-0.05, 0.06)** | **0.01 (-0.03, 0.04)** | **0.01 (-0.03, 0.05)** | **0.01 (-0.04, 0.06)** | **0.01 (-0.03, 0.04)** | **0.75** |
|  | | | | | | |

Presented as mean (95% confidence interval).

** p-value for interaction between gestational age and maternal HIV infection.

˟ Mean difference in growth velocity increments between HIV-positive mothers and HIV-negative mothers at specified gestational ages. † Values are geometric means.

^††^Confidence interval for geometric mean difference.

^†††^ p-values of log-transformed EFW models.

Z-scores calculated with reference to INTERGROWTH-21st foetal growth standards.

**Supplementary Table 7.2 Adjusted mean growth velocity increments and z-scores at different gestational ages by maternal HIV status.**

|  | **19 weeks** | **24 weeks** | **29 weeks** | **34 weeks** | **Overall difference by HIV status** | **p*** | **p_int_**** |
| --- | --- | --- | --- | --- | --- | --- | --- |
| **Head circumference increments** | | | | |  |  |  |
| **HIV negative** | |  |  |  |  |  |  |
| Mean (mm/week) | 12.01 (11.87, 12.14) | 10.73 (10.62, 10.84) | 8.80 (8.69, 8.91) | 6.07 (5.91, 6.23) |  |  |  |
| Mean (Z-score) | -0.08 (-0.22, 0.05) | -0.35 (-0.44, -0.26) | -0.29 (-0.36, -0.21) | -0.02 (-0.12, 0.08) |  |  |  |
| **HIV positive** | |  |  |  |  |  |  |
| Mean (mm/week) | 11.94 (11.78, 12.09) | 10.66 (10.53, 10.80) | 8.73 (8.60, 8.86) | 6.00 (5.82, 6.18) |  |  |  |
| Mean (Z-score) | -0.15 (-0.31, 0.00) | -0.41 (-0.52, -0.29) | -0.33 (-0.43, -0.24) | -0.07 (-0.18, 0.04) |  |  |  |
| **Mean difference˟** | **-0.07 (-0.27, 0.14)** | **-0.07 (-0.24, 0.11)** | **-0.07 (-0.24, 0.10)** | **-0.07 (-0.31, 0.17)** | **-0.07 (-0.24, 0.10)** | **0.42** | **0.92** |
| **Biparietal diameter increments** | | | | |  |  |  |
| **HIV negative** | |  |  |  |  |  |  |
| Mean (mm/week) | 3.25 (3.19, 3.30) | 3.04 (3.00, 3.08) | 2.56 (2.52, 2.60) | 1.79 (1.74, 1.84) |  |  |  |
| Mean (Z-score) | 0.01 (-0.11, 0.14) | -0.20 (-0.28, -0.11) | -0.28 (-0.37, -0.18) | -0.21 (-0.32, -0.11) |  |  |  |
| **HIV positive** | |  |  |  |  |  |  |
| Mean (mm/week) | 3.24 (3.18, 3.30) | 3.03 (2.98, 3.07) | 2.55 (2.50, 2.60) | 1.78 (1.72, 1.83) |  |  |  |
| Mean (Z-score) | -0.01 (-0.15, 0.13) | -0.22 (-0.32, -0.11) | -0.30 (-0.41, -0.19) | -0.24 (-0.36, -0.12) |  |  |  |
| **Mean difference˟** | **-0.01 (-0.09, 0.07)** | **-0.01 (-0.07, 0.05)** | **-0.01 (-0.07, 0.05)** | **-0.01 (-0.09, 0.06)** | **-0.01 (-0.06, 0.04)** | **0.71** | **0.10** |
| **Abdominal circumference** | | | |  |  |  |  |
| **HIV negative** | |  |  |  |  |  |  |
| Mean (mm/week) | 10.89 (10.67, 11.12) | 10.47 (10.30, 10.63) | 10.04 (9.88, 10.21) | 9.62 (9.40, 9.84) |  |  |  |
| Mean (Z-score) | -0.30 (-0.47, -0.14) | -0.18 (-0.28, -0.08) | -0.12 (-0.20, -0.03) | -0.05 (-0.14, 0.04) |  |  |  |
| **HIV positive** | |  |  |  |  |  |  |
| Mean (mm/week) | 11.08 (10.83, 11.33) | 10.66 (10.46, 10.86) | 10.24 (10.04, 10.44) | 9.81 (9.56, 10.07) |  |  |  |
| Mean (Z-score) | -0.17 (-0.35, 0.02) | -0.06 (-0.19, 0.06) | -0.02 (-0.12, 0.08) | 0.03 (-0.07, 0.13) |  |  |  |
| **Mean difference˟** | **0.19 (-0.15, 0.53)** | **0.19 (-0.07, 0.45)** | **0.19 (-0.07, 0.45)** | **0.19 (-0.15, 0.53)** | **0.19 (-0.06, 0.44)** | **0.13** | **0.21** |
| **Femur length** | | |  |  |  |  |  |
| **HIV negative** | |  |  |  |  |  |  |
| Mean (mm/week) | 2.90 (2.86, 2.94) | 2.46 (2.43, 2.48) | 2.10 (2.07, 2.12) | 1.79 (1.76, 1.83) |  |  |  |
| Mean (Z-score) | 0.00 (-0.14 - 0.14) | -0.08 (-0.17 - 0.01) | -0.05 (-0.14 - 0.03) | 0.12 (0.03 - 0.21) |  |  |  |
| **HIV positive** | |  |  |  |  |  |  |
| Mean (mm/week) | 2.90 (2.85, 2.94) | 2.45 (2.42, 2.49) | 2.09 (2.06, 2.13) | 1.79 (1.75, 1.83) |  |  |  |
| Mean (Z-score) | -0.01 (-0.16 - 0.15) | -0.08 (-0.19 - 0.03) | -0.06 (-0.16 - 0.04) | 0.11 (0.01 - 0.22) |  |  |  |
| **Mean difference˟** | **0.00 (-0.06, 0.06)** | **0.00 (-0.04, 0.04)** | **0.00 (-0.05, 0.04)** | **0.00 (-0.06, 0.05)** | **0.00 (-0.04, 0.04)** | **0.94** | **0.28** |
|  | | | | | | | |

Presented as mean (95% confidence interval).

Estimates adjusted for maternal age, baseline BMI, smoking, alcohol, parity, marital status, occupation, education, wealth index score, history of adverse perinatal outcomes and foetal sex.

** p-value for interaction between gestational age and maternal HIV infection.

˟ Mean difference in growth velocity increments between HIV-positive mothers and HIV-negative mothers at specified gestational ages.

^†^ Values are geometric means.

^††^ Confidence interval for geometric mean difference.

^†††^ p-values of log-transformed EFW models.

Z-scores calculated with reference to INTERGROWTH-21^st^ foetal growth standards.

**Supplementary Table 8. Sensitivity analyses of LMM with highest quality ultrasound images**

|  | **Overall mean difference (95% CI)** | **p-value*** |
| --- | --- | --- |
| **Growth measures** |  |  |
| Head circumference (mm) | -0.16 (-1.35, 1.04) | 0.80 |
| Biparietal diameter (mm) | -0.03 (-0.43, 0.38) | 0.90 |
| Abdominal circumference (mm) | -0.20 (-1.65, 1.25) | 0.79 |
| Femur length (mm) | 0.08 (-0.23, 0.40) | 0.62 |
| Estimated foetal weight (grams) ^†^ | 1.01 (0.99, 1.02) ^††^ | 0.54 ^†††^ |
| **Growth velocity increments** |  |  |
| Head circumference increments (mm/week) | -0.12 (-0.32, 0.08) | 0.24 |
| Biparietal diameter increments (mm/week) | -0.03 (-0.10, 0.03) | 0.32 |
| Abdominal circumference(mm/week) | 0.18 (-0.11, 0.48) | 0.23 |
| Femur length (mm/week) | -0.01 (-0.05, 0.04) | 0.82 |
|  | | |

Estimates adjusted for maternal age, baseline BMI, smoking, alcohol, parity, marital status, wealth index score, occupation, education, history of adverse perinatal outcomes and foetal sex.

*p-value for overall difference in mean growth measures or velocity increments between HIV-positive mothers and HIV-negative mothers.

^†^ Values are geometric means.

^††^ Confidence interval for geometric mean difference.

^†††^ p-value of log-transformed EFW models.

**Supplementary Table 9. Association of maternal HIV infection with in-utero SGA and VSGA at last antenatal ultrasound scan before delivery.**

| **Small-for-gestational age (SGA)** | | | | |
| --- | --- | --- | --- | --- |
|  | **Unadjusted OR (95% CI)** | **P-value** | **Adjusted OR (95% CI) *** | **P-value** |
| **Maternal HIV status** |  |  |  |  |
| HIV negative | 1 | ·· | 1 | ·· |
| HIV positive | 0.73(0.50, 1.06) | 0.10 | 0.70(0.44, 1.11) | 0.13 |
| **Very-small-for-gestational age (VSGA)** | | | |  |
|  | **Unadjusted OR (95% CI)** | **P-value** | **Adjusted OR (95% CI) *** | **P-value** |
| **Maternal HIV status** |  |  |  |  |
| HIV negative | 1 | ·· | 1 | ·· |
| HIV positive | 0.74(0.44, 1.25) | 0.26 | 0.69(0.35, 1.30) | 0.24 |

* Adjusted for maternal age, baseline BMI, smoking, alcohol, parity, marital status, wealth index score, occupation, education and history of adverse perinatal outcomes.

SGA and VSGA defined as estimated foetal weight below 10^th^ and 3^rd^ centiles, respectively, with reference to INTERGROWTH 21^st^ foetal standards.

**Supplementary Table 10. Association of maternal HIV infection with SGA and VSGA at birth**

| **Small-for-gestational age (SGA)** | | | | |
| --- | --- | --- | --- | --- |
|  | **Unadjusted OR (95% CI)** | **P-value** | **Adjusted OR (95% CI) *** | **P-value** |
| **Maternal HIV status** |  |  |  |  |
| HIV negative | 1 | ·· | 1 | ·· |
| HIV positive | 1.51(0.99, 2.31) | 0.06 | 1.56(0.93, 2.62) | 0.09 |
| **Very-small-for-gestational age (VSGA)** | | | |  |
|  | **Unadjusted OR (95% CI)** | **P-value** | **Adjusted OR (95% CI) *** | **P-value** |
| **Maternal HIV status** |  |  |  |  |
| HIV negative | 1 | ·· | 1 | ·· |
| HIV positive | 1.48(0.71, 3.09) | 0.30 | 1.78(0.69, 4.61) | 0.23 |

* Adjusted for maternal age, baseline BMI, smoking, alcohol, parity, marital status, wealth index score, occupation, education and history of adverse perinatal outcomes.

SGA and VSGA defined as birthweight below 10^th^ and 3^rd^ centiles, respectively, with reference to INTERGROWTH 21^st^ newborn standards.

# **Supplementary Table 11. Adjusted mean growth measures and z-scores at different gestational ages for PWHIV who received EFV-based ART compared with HIV negative women.**

|  | | **16 weeks** | | **21 weeks** | **26 weeks** | **31 weeks** | **36 weeks** | **Overall difference by HIV status** | **p*** |
| --- | --- | --- | --- | --- | --- | --- | --- | --- | --- |
| **Head circumference** |  | |  | |  |  |  |  |  |
| **HIV negative** | |  | |  |  |  |  |  |  |
| Mean (mm) | | 122.99 (122.18, 123.79) | | 182.97 (182.31, 183.62) | 237.58 (236.77, 238.39) | 282.67 (281.68, 283.67) | 314.83 (313.55, 316.11) |  |  |
| Mean (Z-score) | | 0.01 (-0.12, 0.14) | | -0.20 (-0.29, -0.11) | -0.34 (-0.43, -0.25) | -0.42 (-0.53, -0.32) | -0.42 (-0.54, -0.31) |  |  |
| **PWHIV on EFV-based ART** | |  | |  |  |  |  |  |  |
| Mean (mm) | | 122.69 (121.47, 123.92) | | 182.68 (181.54, 183.82) | 237.29 (236.05, 238.52) | 282.38 (281.01, 283.75) | 314.54 (312.96, 316.12) |  |  |
| Mean (Z-score) | | -0.03 (-0.23, 0.16) | | -0.24 (-0.39, -0.09) | -0.37 (-0.52, -0.23) | -0.45 (-0.60, -0.31) | -0.45 (-0.60, -0.30) |  |  |
| Mean difference˟ | | -0.29 (-1.76, 1.17) | | 0.25 (-1.07, 1.56) | -0.29 (-1.77, 1.18) | -0.29 (-1.99, 1.40) | -0.29 (-2.33, 1.74) | -0.29 (-1.61, 1.03) | 0.66 |
| **Biparietal diameter** | |  | |  |  |  |  |  |  |
| **HIV negative** | |  | |  |  |  |  |  |  |
| Mean (mm) | | 34.93 (34.65, 35.21) | | 51.56 (51.34, 51.79) | 66.87 (66.59, 67.14) | 79.72 (79.39, 80.06) | 89.21 (88.78, 89.64) |  |  |
| Mean (Z-score) | | -0.37 (-0.52, -0.23) | | -0.06 (-0.16, 0.04) | -0.26 (-0.36, -0.16) | -0.54 (-0.65, -0.44) | -0.59 (-0.71, -0.46) |  |  |
| **PWHIV on EFV-based ART** | |  | |  |  |  |  |  |  |
| Mean (mm) | | 34.67 (34.25, 35.10) | | 51.31 (50.91, 51.70) | 66.61 (66.18, 67.04) | 79.46 (79.00, 79.93) | 88.95 (88.41, 89.49) |  |  |
| Mean (Z-score) | | -0.51 (-0.73, -0.29) | | -0.17 (-0.33, 0.00) | -0.35 (-0.51, -0.20) | -0.63 (-0.77, -0.48) | -0.66 (-0.82, -0.51) |  |  |
| Mean difference˟ | | -0.26 (-0.77, 0.25) | | 0.09 (-0.37, 0.55) | -0.26 (-0.77, 0.25) | -0.26 (-0.83, 0.32) | -0.26 (-0.95, 0.43) | -0.25 (-0.72, 0.20) | 0.27 |
| **Abdominal circumference** | |  | |  |  |  |  |  |  |
| **HIV negative** | |  | |  |  |  |  |  |  |
| Mean (mm) | | 100.48 (99.41, 101.54) | | 155.35 (154.51, 156.20) | 208.37 (207.21, 209.53) | 259.09 (257.58, 260.59) | 307.06 (305.07, 309.04) |  |  |
| Mean (Z-score) | | -0.50 (-0.70, -0.31) | | -0.41 (-0.52, -0.31) | -0.39 (-0.50, -0.28) | -0.37 (-0.49, -0.26) | -0.34 (-0.46, -0.22) |  |  |
| **PWHIV on EFV-based ART** | |  | |  |  |  |  |  |  |
| Mean (mm) | | 100.37 (98.81, 101.94) | | 155.25 (153.81, 156.69) | 208.27 (206.61, 209.93) | 258.98 (257.07, 260.90) | 306.96 (304.66, 309.26) |  |  |
| Mean (Z-score) | | -0.52 (-0.81, -0.23) | | -0.42 (-0.60, -0.25) | -0.40 (-0.56, -0.24) | -0.38 (-0.53, -0.23) | -0.35 (-0.48, -0.21) |  |  |
| Mean difference˟ | | -0.10 (-2.00, 1.79) | | 0.30 (-1.37, 1.98) | -0.10 (-2.13, 1.92) | -0.10 (-2.53, 2.33) | -0.10 (-3.14, 2.94) | -0.10 (-1.76, 1.56) | 0.9 |
| **Femur length** | |  | |  |  |  |  |  |  |
| **HIV negative** | |  | |  |  |  |  |  |  |
| Mean (mm) | | 19.39 (19.17, 19.60) | | 34.08 (33.89, 34.27) | 46.57 (46.35, 46.80) | 57.25 (56.99, 57.52) | 66.31 (65.98, 66.65) |  |  |
| Mean (Z-score) | | -0.05 (-0.19, 0.08) | | 0.01 (-0.09, 0.12) | -0.08 (-0.19, 0.03) | -0.11 (-0.23, 0.00) | -0.03 (-0.15, 0.09) |  |  |
| **PWHIV on EFV-based ART** | |  | |  |  |  |  |  |  |
| Mean (mm) | | 19.43 (19.10, 19.77) | | 34.12 (33.80, 34.45) | 46.62 (46.27, 46.97) | 57.30 (56.92, 57.68) | 66.36 (65.93, 66.79) |  |  |
| Mean (Z-score) | | -0.02 (-0.23, 0.19) | | 0.04 (-0.14, 0.22) | -0.06 (-0.23, 0.11) | -0.09 (-0.26, 0.07) | -0.01 (-0.17, 0.14) |  |  |
| Mean difference˟ | | 0.05 (-0.36, 0.45) | | 0.07 (-0.31, 0.44) | 0.05 (-0.37, 0.46) | 0.05 (-0.42, 0.51) | 0.05 (-0.50, 0.59) | 0.05 (-0.33, 0.42) | 0.81 |
| **Estimated foetal weight** | |  | |  |  |  |  |  |  |
| **HIV negative** | |  | |  |  |  |  |  |  |
| Mean (grams) | | 138.88 (137.19, 140.58) | | 369.02 (365.23, 372.85) | 816.58 (807.49, 825.77) | 1534.59 (1515.45, 1553.98) | 2464.55 (2427.13, 2502.54) |  |  |
| Mean (Z-score) | | -0.25 (-0.38, -0.12) | | -0.36 (-0.48, -0.25) | -0.42 (-0.53, -0.30) | -0.44 (-0.55, -0.32) | -0.45 (-0.58, -0.32) |  |  |
| **PWHIV on EFV-based ART** | |  | |  |  |  |  |  |  |
| Mean (grams) | | 138.89 (136.26, 141.58) | | 369.06 (362.46, 375.77) | 816.66 (801.66, 831.94) | 1534.75 (1505.31, 1564.78) | 2464.81 (2413.04, 2517.70) |  |  |
| Mean (Z-score) | | -0.25 (-0.45, -0.05) | | -0.36 (-0.57, -0.16) | -0.42 (-0.61, -0.23) | -0.44 (-0.62, -0.26) | -0.45 (-0.63, -0.27) |  |  |
| Mean difference˟ | | 0.01 (-2.78, 2.81) | | 0.04 (-2.76, 2.84) | 0.09 (-2.71, 2.88) | 0.16 (-2.64, 2.96) | 0.26 (-2.54, 3.07) | 1.00 (0.98, 1.02) | 0.99 |

Presented as predicted mean (95% confidence interval).

Estimates adjusted for maternal age, baseline BMI, smoking, alcohol, parity, marital status, occupation, education, wealth index score, history of adverse perinatal outcomes and foetal sex.

* p-value for overall difference in mean growth measures between PWHIV who received EFV-based ART compared with HIV negative women.

˟ mean difference in growth measures between PWHIV who received EFV-based ART compared with HIV negative women.at specified gestational ages.

Z-scores calculated with reference to INTERGROWTH-21st foetal growth standards.

| Supplementary Table 12. Adjusted mean growth measures and z-scores at different gestational ages for PWHIV by timing of ART initiation. | | | | | | | | |
| --- | --- | --- | --- | --- | --- | --- | --- | --- |
|  | 16 weeks | 21 weeks | 26 weeks | 31 weeks | 36 weeks | | P^*^ | P_int_^**^ |
| **Head circumference (mm)** | | |  |  |  |  | |  |
| **Preconception** |  |  |  |  |  | | |  |
| Mean (mm) | 122.6 (120.7 - 124.5) | 182.3 (180.6 - 184.0) | 236.6 (234.7 - 238.5) | 281.2 (279.0 - 283.5) | 312.8 (310.0 - 315.6) | | |  |
| Mean (Z-score) | -0.05 (-0.36 - 0.25) | -0.28 (-0.50 - -0.06) | -0.45 (-0.68 - -0.23) | -0.58 (-0.82 - -0.33) | -0.61 (-0.87 - -0.35) | | |  |
| **Antenatal** |  |  |  |  |  | | |  |
| Mean (mm) | 123.6 (122.1 - 125.2) | 183.4 (182.1 - 184.7) | 237.6 (236.0 - 239.3) | 282.3 (280.3 - 284.3) | 312.8 (310.0 - 315.6) | | |  |
| Mean (Z-score) | 0.11 (-0.14 - 0.37) | -0.15 (-0.32 - 0.02) | -0.33 (-0.52 - -0.14) | -0.46 (-0.68 - -0.25) | -0.61 (-0.87 - -0.35) | | |  |
| Mean difference ˟ | 1.05 (-1.44 - 3.54) | -0.20 (-2.32 - 1.93) | 1.05 (-1.48 - 3.58) | 1.05 (-1.98 - 4.08) | 1.05 (-2.80 - 4.90) | 0.363 | |  |
| **Biparietal diameter (mm)** | |  |  |  |  |  | |  |
| **Preconception** |  |  |  |  |  | | |  |
| Mean (mm) | 34.6 (33.9 - 35.3) | 51.1 (50.5 - 51.8) | 66.4 (65.6 - 67.1) | 79.2 (78.3 - 80.0) | 88.6 (87.6 - 89.7) | | |  |
| Mean (Z-score) | -0.56 (-0.92 - -0.19) | -0.25 (-0.51 - 0.02) | -0.45 (-0.70 - -0.19) | -0.72 (-0.99 - -0.46) | -0.75 (-1.04 - -0.46) | | |  |
| **Antenatal** |  |  |  |  |  | | |  |
| Mean (mm) | 35.0 (34.5 - 35.6) | 51.6 (51.1 - 52.1) | 66.8 (66.2 - 67.4) | 79.6 (78.9 - 80.3) | 89.1 (88.2 - 90.0) | | |  |
| Mean (Z-score) | -0.32 (-0.62 - -0.03) | -0.06 (-0.27 - 0.15) | -0.29 (-0.50 - -0.07) | -0.58 (-0.81 - -0.35) | -0.62 (-0.88 - -0.35) | | |  |
| Mean difference ˟ | 0.45 (-0.46 - 1.36) | -0.07 (-0.89 - 0.74) | 0.45 (-0.49 - 1.38) | 0.45 (-0.65 - 1.55) | 0.45 (-0.91 - 1.81) | 0.306 | |  |
| **Abdominal circumference(mm)** | | |  |  |  |  | |  |
| **Preconception** |  |  |  |  |  | | |  |
| Mean (mm) | 99.9 (97.4 - 102.4) | 153.8 (151.7 - 156.0) | 206.3 (203.7 - 208.9) | 256.9 (253.8 - 260.0) | 305.4 (301.5 - 309.3) | | |  |
| Mean (Z-score) | -0.60 (-1.07 - -0.14) | -0.60 (-0.87 - -0.33) | -0.59 (-0.84 - -0.34) | -0.54 (-0.78 - -0.30) | -0.44 (-0.66 - -0.21) | | |  |
| **Antenatal** |  |  |  |  |  | | |  |
| Mean (mm) | 102.6 (100.6 - 104.7) | 156.5 (154.9 - 158.2) | 209.0 (206.8 - 211.2) | 259.6 (256.8 - 262.4) | 308.1 (304.5 - 311.7) | | |  |
| Mean (Z-score) | -0.11 (-0.49 - 0.27) | -0.27 (-0.47 - -0.06) | -0.33 (-0.54 - -0.12) | -0.33 (-0.55 - -0.12) | -0.28 (-0.49 - -0.06) | | |  |
| Mean difference ˟ | 2.69 (-0.55 - 5.93) | -0.25 (-3.00 - 2.50) | 2.69 (-0.70 - 6.09) | 2.69 (-1.44 - 6.83) | 2.69 (-2.63 - 8.01) | 0.064 | |  |
| **Femur length (mm)** | |  |  |  |  |  | |  |
| **Preconception** |  |  |  |  |  | | |  |
| Mean (mm) | 19.7 (19.2 - 20.1) | 34.2 (33.8 - 34.7) | 46.7 (46.2 - 47.2) | 57.5 (56.9 - 58.1) | 66.7 (66.0 - 67.5) | | |  |
| Mean (Z-score) | 0.11 (-0.18 - 0.41) | 0.10 (-0.14 - 0.34) | -0.02 (-0.27 - 0.24) | -0.02 (-0.29 - 0.24) | 0.12 (-0.16 - 0.40) | | |  |
| **Antenatal** |  |  |  |  |  | | |  |
| Mean (mm) | 19.5 (19.2 - 19.9) | 34.1 (33.8 - 34.5) | 46.6 (46.2 - 47.0) | 57.4 (56.8 - 57.9) | 66.6 (65.9 - 67.3) | | |  |
| Mean (Z-score) | 0.05 (-0.19 - 0.28) | 0.04 (-0.15 - 0.23) | -0.07 (-0.29 - 0.15) | -0.07 (-0.31 - 0.17) | 0.08 (-0.18 - 0.34) | | |  |
| Mean difference ˟ | -0.11 (-0.72 - 0.50) | -0.05 (-0.60 - 0.50) | -0.11 (-0.78 - 0.57) | -0.11 (-0.93 - 0.71) | -0.11 (-1.15 - 0.93) | 0.710 | |  |
| **Estimated foetal weight (grams)** | | |  |  |  |  | |  |
| **Preconception** |  |  |  |  |  | | |  |
| Mean (mm) | 139.0 (135.6 - 142.5) | 366.0 (357.7 - 374.5) | 805.5 (785.4 - 826.1) | 1511.8 (1470.2 - 1554.7) | 2435.2 (2356.0 - 2516.9) | | |  |
| Mean (Z-score) | -0.24 (-0.50 - 0.02) | -0.46 (-0.72 - -0.20) | -0.56 (-0.82 - -0.30) | -0.58 (-0.83 - -0.32) | -0.55 (-0.82 - -0.28) | | |  |
| **Antenatal** |  |  |  |  |  | | |  |
| Mean (mm) | 141.6 (138.7 - 144.4) | 372.7 (366.1 - 379.4) | 820.4 (803.5 - 837.6) | 1539.7 (1503.1 - 1577.2) | 2480.1 (2407.2 - 2555.2) | | |  |
| Mean (Z-score) | -0.05 (-0.26 - 0.16) | -0.25 (-0.45 - -0.06) | -0.37 (-0.58 - -0.16) | -0.41 (-0.63 - -0.19) | -0.40 (-0.65 - -0.15) | | |  |
| Mean difference ˟ | 2.56 (-0.24 - 5.37) | 6.75 (3.95 - 9.55) | 14.86 (12.05 - 17.66) | 27.89 (25.08 - 30.69) | 44.92 (42.10 - 47.73) | 0.237 | |  |

Presented as predicted mean (95% confidence interval).

Estimates adjusted for maternal age, baseline BMI, smoking, alcohol, parity, marital status, occupation, education, wealth index score, history of adverse perinatal outcomes and foetal sex.

* p-value for overall difference in mean growth measures between PWHIV by timing of ART initiation.

˟ mean difference in growth measures between PWHIV initiated ART by timing of ART initiation at specified gestational ages.

Z-scores calculated with reference to INTERGROWTH-21st foetal growth standards.

# **Supplementary Table 13. Linear mixed effects models specifications**

| Head circumference (HC)  y_i_ = β_0_ + (β_1_) t_i_^2^ + (β_2_) t_i_^2^ ln(t_i_) + e_0i_ + (e_1i_) t_i_ |
| --- |
| Abdominal circumference (AC)  y_i_ = β_0_ + (β_1_) t_i_ + (β_2_) t_i_^3^ + e_0i_ + (e_1i_) t_i_ |
| Biparietal diameter (BPD)  y_i_ = β_0_ + (β_1_) t_i_^2^ + (β_2_) t_i_^2^ ln(t_i_) + e_0i_ + (e_1i_) t_i_ |
| Femur length (FL)  y_i_ = β_0_ + (β_1_) t_i_^1/2^ + (β_2_) t_i_^3^ + e_0i_ + (e_1i_) t_i_ |
| Estimated foetal weight (EFW)  ln (y_i_) = β_0_ + (β_1_) t_i_^1/2^ + (β_2_) t_i_^3^ + e_0i_ + (e_1i_) t_i_ |
| Head circumference increments (HC_inc_)  y_i_ = β_0_ + (β_1_) t_i_^3^ + e_0i_ + (e_1i_) t_i_ |
| Abdominal circumference increments (AC_inc_)  y_i_ = β_0_ + (β_1_) t_i_ + e_0i_ + (e_1i_) t_i_ |
| Biparietal diameter increments (BPD_inc_)  y_i_ = β_0_ + (β_1_) t_i_^2^ + e_0i_ + (e_1i_) t_i_ |
| Femur Length increments (FL_inc_)  y_i_ = (β_1_) ln(t_i_) + e_0i_ + (e_1i_) t_i_ |

y_i_ represents the value of head circumference, abdominal circumference, biparietal diameter, femur length, log-estimated foetal weight, head circumference increments, abdominal circumference increments, biparietal diameter increments or femur length increments for the i^th^ individual at gestational age t_i_.

β_0_ represent the average intercept for each growth measure at t_i_ = 0.

β_1_ and β_2_ represent the coefficients for each gestational age term.

e_0i_ represents the random intercept term for the i^th^ individual and e_1i_ is the random slope for the ith individual.

# **Supplementary Table 14. Outliers**

| **Participant ID** | **Maternal HIV status** | **Follow up scan** | **Gestational age (weeks)** | **Abdominal circumference (mm)** | **Abdominal circumference (z-score)** | **Biparietal diameter (mm)** | **Biparietal diameter (z-score)** | **Head circumference (mm)** | **Head circumference (z-score)** | **Femur length (mm)** | **Femur length (z-score)** | **Pregnancy outcome** | **Birthweight (grams)** |
| --- | --- | --- | --- | --- | --- | --- | --- | --- | --- | --- | --- | --- | --- |
| 14-10109 | Positive | 1 | 16.9 | 8.2 | -5.2 |  |  |  |  |  |  | Live birth | 2585 |
| 14-10139 ^˟^ | Negative | 1 | 11.7 | 242.4 | * | 71.1 | * | 259.6 | * | 51.0 | * | Live birth | 2785 |
| 14-10111 ^§^ | Negative | 1 | 21.3 |  |  | 3.9 | -5.6 |  |  | 2.3 | -6.3 | Live birth | 3140 |
| 14-10141 | Negative | 1 | 18.1 |  |  | 2.6 | -7.6 |  |  |  |  | Live birth | 2480 |
| 14-10363 | Negative | 1 | 15.9 |  |  |  |  |  |  | 1.0 | -5.5 | Miscarriage |  |
| 14-10111 ^§^ | Negative | 2 | 25.4 |  |  |  |  |  |  | 3.4 | -5.8 | Live birth | 3140 |
| 14-10363 | Negative | 2 | 21.0 |  |  |  |  |  |  | 1.4 | -11.1 | Miscarriage |  |
| 14-10455 | Positive | 2 | 21.1 |  |  |  |  |  |  | 2.5 | -5.2 | Stillbirth |  |
| 14-10235 | Negative | 3 | 31 | 15.1 | -8.8 |  |  |  |  |  |  | Live birth | 2525 |
| 14-10297 | Negative | 3 | 24.9 | 13.1 | -7.1 |  |  |  |  |  |  | Live birth | 2950 |
| 14-10391 | Negative | 3 | 27.7 |  |  |  |  |  |  | 3.5 | -7.4 | Live birth | 2683 |
| 14-10398 | Negative | 3 | 28.1 |  |  |  |  |  |  | 3.6 | -7.4 | Live birth | 3180 |
| 14-10111 ^§^ | Negative | 3 | 29.1 |  |  |  |  |  |  | 4.2 | -5.4 | Live birth | 3140 |
| 14-10032 | Negative | 4 | 31.6 |  |  |  |  |  |  | 3.7 | -8.8 | Live birth | 2325 |
| 14-10124 | Negative | 5 | 35.3 |  |  |  |  |  |  | 4.5 | -7.7 | Live birth | 3230 |
| 14-10339 | Negative | 5 | 36 | 18.7 | -7.4 |  |  |  |  |  |  | Live birth | 2899 |

* Z-scores not computed as INTERGROWTH 21^st^ standards are not available for measurements of abdominal circumference, biparietal diameter, head circumference and femur length for foetuses before 14 completed weeks (98 days).

˟ Measurements dropped based on visual inspection of exploratory scatter plots with overlying INTERGROWTH 21^st^ standard centiles.

§ Measurements dropped based on normality of other growth measures and growth velocities of the same participant. Antenatal course was uneventful, and birthweight was 3140 grams.
